# Modeling and Analysis of SIRR Model (Ebola Transmission Dynamics Model) with Delay Differential Equation

**DOI:** 10.12688/f1000research.168361.1

**Published:** 2025-09-02

**Authors:** Akinleye Emmanuel Lasekan, Joshua Oluwasegun Agbomola, Kabir Oluwatobi Idowu, Babatunde Ademola Kannike, Esther Oluwatoyin Mulero, Temitope Senami Gandonu, Solari Myrjuari Elee

**Affiliations:** 1 Department of Mathematics, Lagos State University, Ojo, Lagos, Nigeria; 2 Department of Mathematics, Tulane University, New Orleans, Louisiana, USA; 3 Department of Mathematics, Purdue University, West Lafayette, Indiana, USA; 4 Department of Mathematics, University of Delaware, Newark, Delaware, USA

**Keywords:** SIRR model, delay differential equation, Equilibrium point \sep stability, center manifold theory, Bifurcation Analysis

## Abstract

**Background:**

Ebola virus disease (EVD) is a severe and often fatal illness with high transmission potential and recurring outbreaks. Traditional compartmental models often neglect biologically important delays, such as the latent period before an infected individual becomes infectious, limiting their ability to capture real-world epidemic patterns. Including such delays can provide a more accurate understanding of outbreak persistence and control strategies.

**Methods:**

In this study, we develop and analyze a novel deterministic SIRR model that captures the complex transmission dynamics of Ebola by explicitly combining nonlinear incidence rates with a delay differential equation framework. Unlike traditional models, this approach integrates a biologically motivated delay to represent the latent period before infectiousness, providing a more realistic depiction of disease spread. The basic reproduction number (R _0_) is derived using the next-generation matrix, and local stability for disease-free and endemic equilibria is established. Using center manifold theory, we investigate transcritical bifurcation at R _0_ = 1, while Hopf bifurcation analysis determines when delays trigger oscillatory epidemics. Sensitivity analysis identifies parameters most influencing R _0_, and numerical simulations are performed using the fourth-order Runge–Kutta method.

**Results:**

The main novelty of this work lies in its detailed investigation of how delays influence outbreak persistence and can trigger oscillatory epidemics, patterns often observed in practice but rarely captured by classic models. For R _0_< 1, the disease-free equilibrium is locally asymptotically stable; for R _0_> 1, an endemic equilibrium emerges. Increasing delays destabilizes the system, amplifying peak infections, prolonging outbreaks, and producing sustained oscillations. Isolation of recovered individuals (c) significantly reduces R_0, while transmission rate (β), recruitment rate (Λ), and isolation transition rate (ρ) are identified as the most sensitive parameters.

**Conclusions:**

Accounting for delayed recovery dynamics is crucial for accurately predicting outbreak patterns and designing effective interventions. This delay-based, nonlinear-incidence model offers a robust analytical and computational framework for guiding public health strategies, with direct implications for reducing transmission, shortening outbreak duration, and preventing epidemic resurgence.

## 1. Introduction

Epidemiological modeling plays a pivotal role in understanding and controlling the spread of infectious diseases. ^1^ These models enable researchers and policymakers to analyze disease transmission dynamics, predict outbreaks, and design effective intervention strategies. Among the earliest and most influential frameworks is the SIR model, introduced by Kermack and McKendrick in 1927. ^1^ This simple yet profound model divided the population into three compartments: susceptible, infected, and recovered, and demonstrated the concept of a threshold level of population immunity required to stop an epidemic. The SIR model not only explained how epidemics unfold but also underscored the importance of early interventions. Over the decades, it has remained a cornerstone of infectious disease modeling and public health strategies, forming the basis for countless extensions and refinements in mathematical epidemiology. ^1, 2^

In the mid to late 20th century, the field underwent significant expansion, driven by the work of researchers like Anderson and May. ^3^ They broadened the scope of epidemiological models by incorporating critical factors such as heterogeneous mixing patterns, disease vectors, and variable transmission rates. These innovations made the models more applicable to real-world scenarios, providing insights into the dynamics of diseases like malaria, HIV, and schistosomiasis. Their studies emphasized the importance of understanding transmission dynamics, host-vector relationships, and the role of immunity in shaping the persistence and control of endemic diseases. Additionally, they highlighted the potential impact of control measures such as vaccination and vector management on disease eradication efforts. ^4^

Building on these advancements, Hethcote ^5^ made substantial contributions by addressing nonlinear dynamics, seasonality, and spatial heterogeneity in epidemiological models. His work introduced new dimensions to understanding disease outbreaks, particularly in cases where environmental or seasonal factors play a significant role. These refinements underscored the complexity of real-world disease transmission and the need for models capable of capturing multifaceted interactions among biological, social, and environmental variables. The concept of the basic reproduction number R _0_, as discussed by Heesterbeek and Dietz, ^6^ further formalized how to quantify the potential of a pathogen to spread in a population.

The urgency and complexity of Ebola virus outbreaks have driven widespread interdisciplinary research, especially in mathematical modeling, to guide timely and practical intervention strategies. Ebola virus disease (EVD) is particularly challenging due to its high fatality rate, rapid symptom development, and potential for international spread. These factors make it not only a medical emergency but also a complex modeling problem that requires input from biology, sociology, and public health policy. Over the years, scientists have refined models to include not just disease transmission but also the societal, ecological, and decision-making contexts in which these outbreaks unfold. One of the earlier efforts to understand the spread of Ebola was made by Lekone and Finkenstädt. ^7^ They used a stochastic SEIR model, which stands for Susceptible, Exposed, Infectious, and Recovered, to simulate how interventions like isolating patients and tracing their contacts could slow down the spread. What made their approach notable was how they factored in the randomness of real-world disease spread, something that becomes even more important in smaller populations where each case can dramatically change the outbreak's trajectory. Soon after, Ndanguza and colleagues ^8^ focused on data from the 1995 outbreak in the Democratic Republic of Congo. They used statistical tools to dig into what actually happened during that outbreak. This kind of retrospective analysis helped build more accurate models for future use by highlighting how local healthcare quality and contact patterns influenced disease transmission over time. In 2014, Nigeria's experience with Ebola became a model for successful containment. Fasina et al. ^9^ built a model to understand how early diagnosis, rapid response, and effective isolation measures helped stop the virus from spreading widely. Their work showed that local readiness, like good communication and coordination between agencies, can dramatically change the outcome of an epidemic, even in highly populated cities. Agusto et al. ^10, 11^ added a cultural layer to traditional epidemiological models. They looked at how local customs, such as burial practices, affected transmission. For instance, traditional funerals often involve close physical contact with the deceased, which can unknowingly contribute to further infections. Their model demonstrated that overlooking these behaviors could lead to significant underestimation of outbreak severity. This idea was supported by Weitz and Dushoff, ^12^ who explored how the virus can still be contagious even after the host has died, making safe burial practices a critical public health intervention. On a broader scale, Ivorra and colleagues ^13^ developed the Be-CoDiS model, short for Between-Countries Disease Spread. This model wasn't just about one community or one country, it was about how people, goods, and even the virus itself move across borders. Their model helped global health agencies better predict which countries might be at risk next and what steps could be taken to prepare. Berge et al. ^14^ focused on making models that are both simple and useful, especially for countries with limited data and resources. They showed that even basic models could provide valuable guidance if they focused on the right variables, like how long someone remains infectious or how quickly they can be isolated. These small adjustments in the model had a big impact on the accuracy of predictions. Rachah and Torres ^15^ approached the problem using control theory. Their models helped answer questions like: "How much should we spend on vaccinations? When should we focus more on public education?" Their findings suggested that adjusting strategies over time, rather than sticking to a fixed plan, often leads to better health outcomes and a more efficient use of resources. Abo and Smith ^16^ explored what happens when vaccines are either available or unavailable. They built a model that accounted for changes in vaccine supply and how public acceptance might vary. Their work showed that even short interruptions in vaccination campaigns can drastically increase risk, highlighting the importance of consistent public health messaging and resource allocation. At the community level, Marais et al. ^17^ stressed the need for cultural sensitivity in designing infection control programs. Their research showed that people are more likely to follow health guidelines when local leaders and traditions are involved in planning and communication. For example, religious leaders could help shape messages in a way that respects local customs while still promoting safe behavior. On the ecological side, Leroy et al. ^18^ discovered that fruit bats likely serve as a natural reservoir for the Ebola virus. This means the virus can survive in these animals without making them sick, only to spill over to humans under certain conditions. Their work reinforced the idea that human encroachment into wildlife habitats, like forests, isn't just an environmental issue; it's a public health risk too. Burkett-Cadena and Vittor ^19^ expanded on this by modeling how environmental changes, like deforestation, bring humans and wildlife into closer contact. Their models demonstrated how clearing forests or altering ecosystems can create new opportunities for viruses to jump from animals to people, making future outbreaks more likely. In terms of data-driven modeling, Kucharski et al. ^20^ used real-time data from Sierra Leone to adjust their models as the epidemic unfolded. This approach helped public health officials make quicker and better-informed decisions, such as when to deploy more resources or change intervention strategies. Their method marked a shift toward more responsive, real-time modeling that adapts to the outbreak as it evolves. Nishiura and Chowell ^21^ brought all these ideas together in a comprehensive review. They evaluated dozens of different models to see what worked and what didn't. One of their key takeaways was that modeling accuracy improves dramatically when uncertainty, cultural diversity, and differences in healthcare access are taken seriously. They also called for better data-sharing practices to help researchers and governments act more cohesively during crises. Altogether, these studies reflect just how multifaceted Ebola modeling has become. Today's models aim to do more than just predict numbers, they try to capture the human, social, and ecological realities behind those numbers. Whether it's the behavior of a virus, a person, or a government, integrating all these layers gives us a better shot at preventing the next outbreak. The evolution of these modeling approaches shows that science and society must work hand-in-hand to effectively combat emerging infectious diseases. Agbomola and Loyinmi ^22, 23^ analyzed the impact of human-bat interactions and control strategies on Ebola transmission using optimal control models, emphasizing the role of vector management and safe burial practices. Legrand et al. ^24^ provided critical insights into the effectiveness of public health interventions such as isolation, contact tracing, and the promotion of safe burial practices, which are crucial in breaking chains of transmission during outbreaks. Pandey et al. ^25^ explored the combined effects of vaccination campaigns, public awareness, and behavioral changes in reducing the spread of Ebola, offering a framework for understanding how early interventions could mitigate future outbreaks. Rivers et al. ^23, 26^ demonstrated the power of real-time modeling in predicting outbreak trajectories, providing policymakers with tools to allocate resources effectively during the West African Ebola epidemic. Similarly, Althaus ^27, 28^ employed dynamic transmission models to estimate key epidemiological parameters, such as the basic reproduction number R _0_, for different stages of the epidemic. These models offered vital insights into the rate of spread and the effectiveness of interventions over time. Chretien et al. ^29^ underscored the role of environmental and ecological factors in Ebola outbreaks, highlighting how climatic conditions and land-use patterns influence disease emergence and persistence. While traditional models like the SIR and its derivatives provided a robust framework, they initially lacked the capacity to account for delays inherent in biological and societal processes. Delays, such as the incubation period of a disease, the time lag in symptom onset, or delays in implementing public health interventions, are crucial to accurately capturing disease dynamics. Addressing this limitation, delay differential equations (DDEs) emerged as a powerful mathematical tool to incorporate time-dependent processes into epidemiological models. Early contributions by Cooke and van den Driessche ^30^ demonstrated the utility of integrating delays into compartmental models, allowing for more realistic representations of disease progression and intervention outcomes. These models could, for example, simulate the effects of latent periods or the time-dependent impact of treatment protocols. Epidemiological modeling has long relied on ordinary differential equations (ODEs) to represent the dynamic spread of infectious diseases. These models, typified by the classical SIR framework, offer mathematical simplicity and analytical tractability. However, they inherently assume that all changes in a population's disease status occur instantaneously, overlooking the biological reality that many processes in disease transmission involve time delays. For instance, incubation periods, delays in symptom onset, and the lag between infection and the implementation of control measures are all time-dependent factors that shape disease dynamics in fundamental ways. ^5^ By treating such processes as instantaneous, ODE-based models risk oversimplifying complex epidemiological phenomena. Delay differential equations (DDEs) address this critical limitation by explicitly incorporating time lags into the modeling framework. ^30– 32^ This refinement allows for the representation of delayed infection responses, delayed treatment outcomes, and other temporally structured processes, offering a more biologically realistic depiction of disease progression.

Beyond epidemiology, the mathematical foundation of DDEs is well-established in dynamical systems theory, with results on stability analysis, bifurcation theory, and solution behavior extensively developed by researchers such as Hale and Lunel ^33^ and Beretta and Kuang. ^34^ These advances provide rigorous tools for understanding threshold phenomena, long-term behavior, and the conditions under which diseases can be controlled or eradicated. The flexibility of DDEs also extends to computational modeling. Shampine and Thompson ^35^ have shown how modern numerical methods can efficiently solve DDE systems, enabling the simulation of complex scenarios with time delays, such as delayed response to interventions or the accumulation of immunity in a population over time.

In addition, while ODE models offer foundational insights, DDEs provide a more accurate, realistic, and mathematically robust framework for modeling infectious diseases. ^36, 37^ The explicit incorporation of delays bridges the gap between theoretical constructs and the biological realities of disease transmission, offering policymakers and public health experts more reliable tools for predicting outbreaks and designing effective interventions. ^30– 32, 38^

## 2. Model formulation

**Figure 1.**
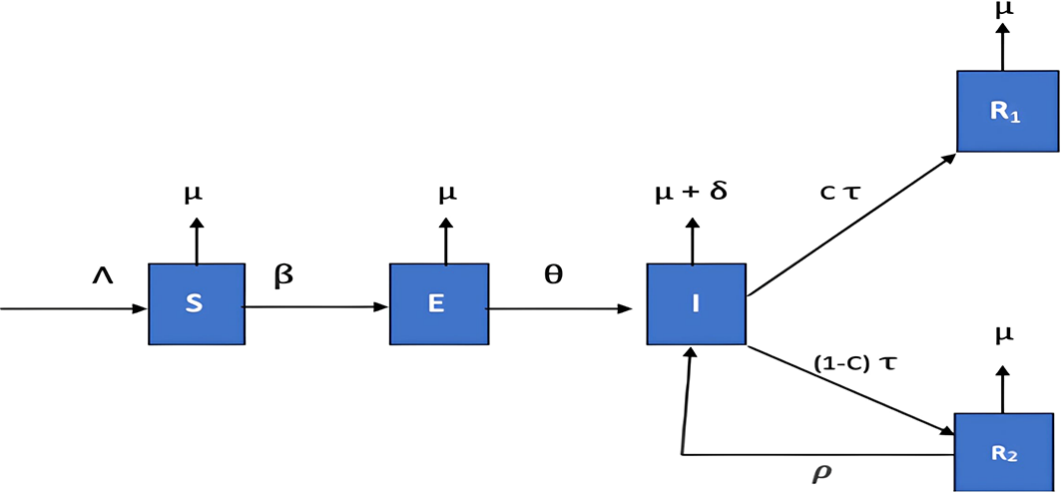
Schematic diagram.

We examine the SEIRR model presented by Ref. 39, whose structure is illustrated in the schematic diagram below Figure 1 and Parameters Description are contained in Table 1. The model focuses on the host compartment of the epidemiological system, where the total population N is divided into distinct epidemiological classes:

• **Susceptible** ( S ): Individuals at risk of infection
• **Exposed** ( E ): Infected individuals not yet infectious
• **Infectious** ( I ): Individuals capable of transmitting the disease

The model further distinguishes between two types of **Recovered Individuals:** Even after recovering from Ebola with medical treatment, patients may still carry traces of the virus in certain parts of their bodies. Research shows the virus can linger in places like semen, eye fluid, breast milk, and spinal fluid - sometimes for weeks or months after symptoms disappear. The subdivision of the Recovered Individuals is:

• **Recovered in Isolation** ( $R_{1}$ ): Individuals who have recovered but remain under medical isolation due to potential viral persistence in body fluids
• **Recovered without Isolation** ( $R_{2}$ ): Individuals who have recovered and are not under isolation protocols

### 2.1 Model without delay differential equation

dSdt=Λ−μS−βSIdEdt=βSI−(μ+θ)EdIdt=θE+ρR2−(μ+δ+cτ+(1−c)τ)IdR1dt=cτI−μR1dR2dt=(1−c)τI−(μ+ρ)R2 (1)

With Initial condition

$$S\left(t\right)\;\geq \;0,\;E\left(t\right)\;\geq \;0,\;I\left(t\right)\;\geq \;0,\;R_{1}\left(t\right)\;\geq \;0,\;R_{2}\left(t\right)\;\geq \;0$$

Where

$$S\left(t\right)+E\left(t\right)+I\left(t\right)+R_{1}\left(t\right)+R_{2}\left(t\right)=N$$

### 2.2 Positivity of the model

*1. Theorem*

All the solutions of the system (1) are non-negative for t≥0 .

Proof Let

$$\hat{y}=\sup\left\{t>0:S\left(t\right)\;\geq \;0,E\left(t\right)\;\geq \;0,I\left(t\right)\;\geq \;0,R\left(t\right)\;\geq \;0,R_{2}\left(t\right)\;\geq \;0,P_{E}\left(t\right)\;\geq \;\theta \right\}$$

then

$$\hat{y}>0$$

From the first equation in (1)

$$\frac{\mathrm{dS}}{\mathrm{dt}}=\Lambda -\mathrm{\mu S}-\mathrm{\beta SI}$$

$$\frac{\mathrm{dS}}{\mathrm{dt}}+\left(\mu +\mathrm{\beta I}\right)S=\Lambda$$

$$\text{IF}=e^{\int \left(\mu +\mathrm{\beta I}\right)\mathrm{dt}}=e^{\int \left(\mu +\mathrm{\beta I}\right)\mathrm{dt}}$$

$$e^{\int \left(\mu +\mathrm{\beta I}\right)\mathrm{dt}}\frac{\mathrm{dS}}{\mathrm{dt}}+e^{\int \left(\mu +\mathrm{\beta I}\right)\mathrm{dt}}\left(\mu +\mathrm{\beta I}\right)S=\Lambda e^{\int \left(\mu +\mathrm{\beta I}\right)\mathrm{dt}}$$

$$\frac{d}{\mathrm{dt}}\left\{Se^{\int \left(\mu +\mathrm{\beta I}\right)\mathrm{dt}}\right\}=\Lambda e^{\int \left(\mu +\mathrm{\beta I}\right)\mathrm{dt}}$$

Let

$$\left(S\left(0\right)=S_{0},E\left(0\right)=E_{0},I\left(0\right)=I_{0},R_{1}\left(0\right)=R_{1_{0}},R_{2}\left(0\right)=R_{2_{0}}\right)$$

$$S\left(t\right)=e^{-\int_{0}^{t}\left(\mu +\mathrm{\beta I}\right)\;\mathrm{dt}}\left(S\left(0\right)+\int_{0}^{t}\Lambda e^{\int_{0}^{s}\left(\mu +\mathrm{\beta I}\right)\;\mathrm{dt}}\;\mathrm{dt}\right)$$

As S(t)≥0 , S is non-negative.

Similarly, we prove that other compartments

$$E\left(t\right),I\left(t\right),R_{1}\left(t\right),R_{2}\left(t\right)$$

are positive

∀t≥0.

### 2.3 Boundedness of the model

*2. Theorem*

The analytic solutions of the system of equations in (1) are bounded.

Proof By summing the system of equations in (1)

$$\frac{\mathrm{dS}}{\mathrm{dt}}+\frac{\mathrm{dE}}{\mathrm{dt}}+\frac{\mathrm{dI}}{\mathrm{dt}}+\frac{dR_{1}}{\mathrm{dt}}+\frac{dR_{2}}{\mathrm{dt}}$$

which simplifies to:

$$\frac{d\left(S+E+I+R_{1}+R_{2}\right)}{\mathrm{dt}}=\Lambda -\mu \left(S+E+I+R_{1}+R_{2}\right)-\mathrm{\delta I}$$ (2)

which gives:

$$\frac{d\left(S+E+I+R_{1}+R_{2}\right)}{\mathrm{dt}}\;\leq \;\Lambda -\mu \left(S+E+I+R_{1}+R_{2}\right)$$

Recall $S+E+I+R_{1}+R_{2}=N$

$$\frac{\mathrm{dN}}{\mathrm{dt}}\;\leq \;\Lambda -\mathrm{\mu N}$$

Integrating:

$$N\left(t\right)\;\leq \;\frac{\Lambda }{\mu }+Ce^{-\mathrm{\mu t}}$$

As t→∞ , $Ce^{-\mathrm{\mu t}}$ decays to zero:

$$N\left(t\right)\;\leq \;\frac{\Lambda }{\mu }$$

Hence:

$$\underset{t\to \infty }{\mathrm{limsup}}\left(S+E+I+R_{1}+R_{2}\right)\;\leq \;\frac{\Lambda }{\mu }$$

The feasible region for the system of equations in (1) is:

$$\Omega =\left\{(S,E,I,R_{1},R_{2});S+E+I+R_{1}+R_{2}\;\leq \;\frac{\Lambda }{\mu },S\;\geq \;0,E\;\geq \;0,I\;\geq \;0,R_{1}\;\geq \;0,R_{2}\;\geq \;0\right\}$$

## 3. Basic Reproductive Number ( $R_{0}$ )

From the system of equation in (1)

dSdt=Λ−μS−βSIdEdt=βSI−(μ+θ)EdIdt=θE+ρR2−(μ+δ+cτ+(1−c)τ)IdR1dt=cτI−μR1dR2dt=(1−c)τI−(μ+ρ)R2 (3)

At DFE

$$\left[S,E,I,R_{1},R_{2}\right]=\left[\frac{\Lambda }{\mu },0,0,0,0\right]$$

$$\mathrm{Let}\;X^{\prime }={\left[E,I,R_{2}\right]}^{T}\;\left(\text{Disease states of infection}\right)$$

$$X^{*}=F\left(X^{*}\right)-V\left(X^{*}\right)$$

$$F=\left[\begin{array}{ccc} 0 & \mathrm{\beta SI} & 0\\ 0 & 0 & 0\\ 0 & 0 & 0 \end{array}\right]$$

$$V=\left[\begin{array}{ccc} \left(\mu +\theta \right)E & 0 & 0\\ -\mathrm{\theta E} & \left(\mu +\delta +c_{1}\tau +\left(1-c\right)\tau \right)I & -\rho R_{2}\\ 0 & -\left(1-c\right)\mathrm{\tau I} & \left(\mu +\rho \right)R_{2} \end{array}\right]$$

$${F\left(x\right)=\frac{\partial F}{\partial x}|}_{\mathrm{at}\;\mathrm{DFE}}$$

$${V\left(x\right)=\frac{\partial V}{\partial x}|}_{\mathrm{at}\;\mathrm{DFE}}$$

$$F\left(x\right)=\left[\begin{array}{ccc} 0 & \mathrm{\beta S} & 0\\ 0 & 0 & 0\\ 0 & 0 & 0 \end{array}\right]=\left[\begin{array}{ccc} 0 & \beta \frac{\Lambda }{\mu } & 0\\ 0 & 0 & 0\\ 0 & 0 & 0 \end{array}\right]$$

$$V\left(x\right)=\left[\begin{array}{ccc} \left(\mu +\theta \right) & 0 & 0\\ 0 & \left(\mu +\delta +c_{1}\tau +\left(1-c\right)\tau \right) & -\rho \\ 0 & -\left(1-c\right)\tau & \left(\mu +p\right) \end{array}\right]$$

$${\mathrm{FV}}^{-1}=\left(\begin{array}{ccc} \frac{\beta \Lambda \theta \left(\mu +\rho \right)}{\mu \left(\theta +\mu \right)\left(\left(\mu +\rho \right)\left(c\tau +\delta +\mu \right)-\left(c-1\right)\mu \tau \right)} & \frac{\beta \Lambda \left(\mu +\rho \right)}{\mu \left(\left(\mu +\rho \right)\left(c\tau +\delta +\mu \right)-\left(c-1\right)\mu \tau \right)} & \frac{\beta \Lambda \rho }{\mu \left(\left(\mu +\rho \right)\left(c\tau +\delta +\mu \right)-\left(c-1\right)\mu \tau \right)}\\ 0 & 0 & 0\\ 0 & 0 & 0 \end{array}\right)$$

$$R_{0}=\frac{\beta \Lambda \theta \left(\mu +\rho \right)}{\mu \left(\theta +\mu \right)\left(\left(\mu +\rho \right)\left(\mathrm{c\tau}+\delta +\mu \right)-\left(c-1\right)\mu \tau \right)}$$

## 4. Model with delay differential equation

It is common to observe that infected individuals do not transmit the infection to others immediately. There is a time delay before any confirmed case can occur after the exposure. This delay is known as the **latent period**. This introduces a gap between exposure and when the infection becomes transmissible. In mathematical form, this latent period is modeled as a time delay in the infection process. Time delays can destabilize the equilibrium point of the system, leading to a **Hopf bifurcation**. This results in periodic oscillations. This motivates the inclusion of the delay in the model reflecting that individuals infected at time $t-\tau_{2}$ can spend at time t .

Let’s take into consideration a delayed SIRR model with a saturation incidence rate and exponential birth rate:

$$\frac{S\left(t-\tau_{2}\right)I\left(t-\tau_{2}\right)e^{-\mu_{2}\tau_{2}}}{1+\mathrm{mI}\left(t-\tau_{2}\right)}$$

where m is a constant related to the saturation effect.

The system of equations below represents the delay differential equations from this model:

dSdt=Λ−μS−βS(t−τ2)I(t−τ2)e−μ2τ21+mI(t−τ2)dIdt=βS(t−τ2)I(t−τ2)e−μ2τ21+mI(t−τ2)+ρR2−(μ+δ+cτ+(1−c)τ)IdR1dt=cτI−μR1dR2dt=(1−c)τI−(μ+ρ)R2 (4)

From the system of equations, we have in (4)

We can see that $R_{1}$ and $R_{2}$ compartments are independent of S and I .

Thus, it’s enough to consider the following reduced system of equations for our study:

dSdt=Λ−μS−βS(t−τ2)I(t−τ2)e−μ2τ21+mI(t−τ2)dIdt=βS(t−τ2)I(t−τ2)e−μτ21+mI(t−τ2)−(μ+δ+cτ+(1−c)τ)I (5)

with initial conditions S(t)≥0,I(t)≥0 .

### 4.1 Equilibrium and stability analysis

The equilibria of system (5) are found by setting the right-hand side of the system to zero. A stable endemic equilibrium point is a fixed point in a system where if the system is slightly disturbed, it will eventually return to that point. Therefore, the equilibrium solutions of a system with a time delay are the same as those of the corresponding system without delay.

### 4.2 Disease Free Equilibrium

The D.F.E can be found by setting the system of equation in 3 to zero. Which is $P=\left\{\frac{\Lambda }{\mu },0\right\}$ i.e. $P=\left\{S_{0},0\right\}$

The basic reproductive number $R_{0}$ : This is the number of secondary infections caused by a single infected individual in a completely susceptible population.

From system (4), we compute $R_{0}$ by setting

$$\frac{\mathrm{dI}}{\mathrm{dt}}\;\geq \;0,\;\text{assuming there is}\;\mathrm{no}\;\text{delay}\;I\left(t\right)\to 0.$$

$$\frac{\mathrm{dI}}{\mathrm{dt}}=\mathrm{\beta SI}+\rho R_{2}-\left(\mu +\delta +\mathrm{c\tau}+\left(1-c\right)\tau \right)I\;\geq \;0$$ (6)

Assuming $R_{2}\to 0$ , we have:

$$\beta S_{0}\;\geq \;\left(\mu +\delta +\mathrm{c\tau}+\left(1-c\right)\tau \right)\Rightarrow \frac{\beta S_{0}}{\mu +\delta +\mathrm{c\tau}+\left(1-c\right)\tau }\;\geq \;1$$

Thus,

$$\frac{\beta \Lambda }{\mu \left(\mu +\delta +\mathrm{c\tau}+\left(1-c\right)\tau \right)}\;\geq \;1$$

Finally, we get:

$$R_{0}=\frac{\beta \Lambda }{\mu \left(\mu +\delta +\mathrm{c\tau}+\left(1-c\right)\tau \right)}$$

*3. Theorem*

The disease-free equilibrium P is locally asymptotically stable if $R_{0}<1$ and unstable if $R_{0}>1$ $\forall \;\tau_{2}\;\geq \;0$

Proof Let F and V be 3×3 matrices representing the infection and transition dynamics, respectively, evaluated at the DFE. Assume that F≥0 (non-negative matrix) and V is a nonsingular M -matrix (i.e., a matrix whose inverse is non-negative and has eigenvalues with positive real parts).

$$J=F-V=\left(\begin{array}{ccc} F_{11}-V_{11} & F_{12}-V_{12} & F_{13}-V_{13}\\ F_{21}-V_{21} & F_{22}-V_{22} & F_{23}-V_{23}\\ F_{31}-V_{31} & F_{32}-V_{32} & F_{33}-V_{33} \end{array}\right)$$

The basic reproductive number

$$R_{0}=\rho \left(FV^{-1}\right)$$

CASE 1 When R ${\;}_{0}$ < 1

We know that F≥0 and V is a non-singular M -matrix. Hence, $R_{0}=\rho \left(FV^{-1}\right)>1$ , and all eigenvalues of (F−V) have negative real parts.

Since $R_{0}=\rho \left(FV^{-1}\right)>1$ , we have that:

$$\left(I-FV^{-1}\right)\;\geq \;0$$

If $\left(V-F\right)\;\geq \;V\left(I-V^{-1}F\right)$ , then ${\left(V-F\right)}^{-1}\;\geq \;{\left(I-V^{-1}F\right)}^{-1}$ $V^{-1}$ . Recall that $V^{-1}\;\;\geq \;\;0$ .

It follows that:

$${\left(V-F\right)}^{-1}\;\geq \;0$$

Therefore, V−F is a non-singular M -matrix, and by the characteristics of non-singular M -matrices, all the eigenvalues of V−F have positive real parts.

Consequently, all eigenvalues of F−V have negative real parts, implying that the DFE is locally asymptotically stable.

CASE 2 When $R_{0}>1$

We can prove that if $R_{0}>1$ , the DFE is unstable. Assume $R_{0}>1$ and suppose by contradiction that all eigenvalues of F−V have non-negative real parts.

λ:(F−V)≥0,∀i,wherei∈ℕ

Since ϵ>0 is arbitrary:

$$\left(I+\mathrm{\epsilon I}\right)-FV^{-1}$$

For any ϵ>0 , this matrix $\left(I+\mathrm{\epsilon I}\right)-FV^{-1}$ remains a non-singular M -matrix. Hence, it has all positive eigenvalues.

Thus, when $R_{0}>1$ , the matrix F−V has at least one eigenvalue with a positive real part, which implies the DFE is unstable.

CASE 3 When $R_{0}=1$

When $R_{0}=1$ we can see that $\beta =\beta^{*}=\frac{\mu \left(\mu +\delta +\mathrm{c\tau}+\left(1-c\right)\tau \right)}{\Lambda }$

When $\beta^{*}$ is the bifurcation parameter, hence the system has a zero eigenvalue (simple eigenvalue) and the other is a negative eigenvalue, i.e. nonhyperbolic equilibrium.

To study the thermal stability of the nonhyperbolic equilibrium, we will need to apply the center manifold theory [Sastry(1999)] ^42^ and Theorem 4.1 of [castillo-chaves and song (2004)] ^40^

To describe this, let’s consider the system a general system for ordinary differential equations.

$$\frac{\mathrm{dx}}{\mathrm{dt}}=f\left(x,\beta^{*}\right),f:{\mathbb{R}}^{n}\times \mathbb{R}\to {\mathbb{R}}^{n}\;\text{and}\;f\in C^{2}\left({\mathbb{R}}^{n}\times \mathbb{R}\right)$$

Let $x_{1}=S,x_{2}=I$ , the system in (5) becomes:

F1=dx1dt=Λ−μx1−β∗x1(t−τ)x2(t−τ)e−μ2τ21+mx2(t−τ)F2=dx2dt=β∗x1(t−τ)x2(t−τ)e−μ2τ21+mx2(t−τ)+ρx4−(μ+δ+cτ+(1−c)τ)x2 (7)

At $R_{0}=1,\beta =\beta^{*}=\frac{\mu \left(\mu +\delta +\mathrm{c\tau}+\left(1-c\right)\tau \right)}{\Lambda }$

at $\mathrm{DFE}\left(x_{1}=\frac{\Lambda }{\mu },x_{2}=0\right)$

The jacobian matrix of the system above around DFE

$$\mathrm{Det}=\left[\begin{array}{cccc} -\mu & & & \frac{-\beta^{*}\Lambda }{\mu }\\ 0 & & & 0 \end{array}\right]$$

Let’s consider the right eigenvector associated with 0 eigenvalue which is $u=\left[u_{1},u_{2}\right]=\left[\frac{-\beta \Lambda }{\mu^{2}},1\right]$ and left eigen vector associated with the zero eigenvalue (simple eigenvalue is $w=\left[w_{1},w_{2}\right]=\left[0,1\right]$ )

When we consider the partial derivative associated with the system.

∂2F1∂x1∂x2=−β∗,∂2F1∂x12=0∂2F2∂x1∂x2=β∗,∂2F2∂x22=−2Λmβ∗μ∂2f2∂x2∂β∗=Λμ

Now we can apply Theorem 4.1[Castillo-chaves and song[2004] ^40^

When the formula for a and b are

a=∑k=1,j=1nwkuiuj∂2fk∂xi∂xj(0,0)b=∑k=1,j=1nwkui∂2k∂x1∂β(0,0)a=∑k=1n∑1=1n∑j=1wkuiuj∂2F2∂x∂y(0,0).=−(2β∗Λ(β∗+μm)μ2)<0

$$b=u_{2}\left(\frac{\Lambda }{\mu }\right)=\frac{\Lambda }{\mu }>0$$

**1. Remarks:**

From Theorem 4.1[Castillo chaves and song [2004]. ^40^ Since a<0 and b>0, the system undergoes a transcritical bifurcation at $R_{0}=1$ as the bifurcation parameter is varied.

### 4.3 Endemic equilibrium

To establish the conditions for the existence of endemic equilibrium $P^{*}=\left(S^{*},I^{*}\right)$ , when the disease remains present in the population, the system of equation (5) is reformulated to derive the fixed points for $S^{*}$ and $I^{*}$ yielding the following results;

(1)
$I^{*}=\frac{\Lambda -\mu s^{*}}{\mu s^{*}m+\beta s^{*}-\Lambda m}$

(2)
$\left\{s^{*}\right\}$

$S^{*}$ is given by the quadratic equation;

$$h_{1}s^{2*}+h_{2}s^{*}+h_{3}=0$$

Where

h1=zμ−μβ−μ2mh2=Λμm+Λβ+μm+ρR2Λm+ρR2βh3=−Λ2m−ρR2Λm−zΛ

Where z=(μ+δ+cτ+(1−c)τ)

By Rene Descarte’s rule of sign we can say that the quadratic equation have a unique positive real root if the following condition holds:

$$h_{1}>0,h_{2}>0,h_{3}<0$$

Local stability of the endemic equilibrium $P^{*}$ is analysed as follows; The characteristic equation of system evaluated at $P^{*}$

The characteristic equation

$$\lambda^{2}+J_{1}\lambda +k_{1}+\left(J_{2}\lambda +k_{2}\right)e^{-\lambda \tau_{2}}$$

When we have

J1=μ+z+βI∗(1+mI∗),k2=mβs∗(1+mI∗)2k1=zμ+zβI∗(1+mI∗)J2=−βs∗(1+mI∗)2

To ensure that the endemic equilibrium $P^{*}$ is locally asymptotically stable, we need both eigenvalues λ to have negative real parts.

According to the routh-Hurwitz criterion, this happens if and only if;

J>0k>0

Hence the theorem:

*4. Theorem*

for $\tau_{2}=0$ the endemic equilibrium $P^{*}=\left(S^{*},I^{*}\right)$ is locally asymptotically stable if both of the following conditions hold simultaneously;

(1)
$\frac{s^{*}}{I^{*}}\;\leq \;\frac{\mu }{z}$

(2)
$\frac{s^{*}}{I^{*}}\;\leq \;I$
J=J1+J2=μ+z+βI∗(1+mI)−βs∗(1+mI)2=μ+z+βI+βmI2∗−βs∗(1+mI)2>0k=k1+k2=zμ+zβI∗(1+mI∗)+μβs∗(1+mI∗)2>0

Hence J,k>0 and satisfies Routh-Hurwitz criterion.

## 5. Hopf Bifurcation analysis

The characteristic equation is given by:

$$\lambda^{2}+J_{1}\lambda +k_{1}+\left(J_{2}\lambda +k_{2}\right)e^{-\lambda \tau_{2}}=0.$$ (8)

Substituting λ=iw :

$$\left(-w^{2}+\mathrm{iw}J_{1}+k_{1}\right)+\left(iJ_{2}+k_{2}\right)\left(\cos\left(w\tau_{2}\right)-i\sin\left(w\tau_{2}\right)\right)=0.$$ (9)

Separating real and imaginary parts:

Real part:−w2+k1+k2cos(wτ2)−wJ2sin(wτ2)=0,Imaginary part:wJ1+wJ2cos(wτ2)−k2sin(wτ2)=0.

Squaring and adding these parts eliminates $\cos\left(w\tau_{2}\right)$ and $\sin\left(w\tau_{2}\right)$ , yielding:

$$w^{4}+\left(J_{1}^{2}+J_{2}^{2}-2k_{1}\right)w^{2}+\left(k_{1}^{2}-k_{2}^{2}\right)=0.$$ (10)

Let $\xi_{1}=w^{2}$ . The equation becomes:

$$\xi_{1}^{2}+P\xi_{1}+\Delta =0,$$ (11)

where:

$$P=J_{1}^{2}+J_{2}^{2}-2k_{1},\;\Delta =k_{1}^{2}-k_{2}^{2}.$$

then we get,

$$\xi_{1}^{2}+P\xi_{1}+\Delta =0.$$

From the equation above

$$\tau_{n}=\frac{1}{w}\arccos\left(\frac{\left(k_{2}-J_{1}J_{2}\right)w^{2}-k_{1}k_{2}}{J_{2}w^{2}+k_{1}^{2}}\right)+\frac{2\mathrm{n\pi}}{w},\;n=0,1,2,\ldots$$

Assuming the discriminant $P^{2}-4\Delta <0$ .

$$\lambda^{2}+J_{1}\lambda +k_{1}+\left(J_{2}\lambda +k_{2}\right)e^{-\lambda \tau_{2}}=0$$

Differentiate with respect to $\tau_{2}$

$$2\lambda \frac{\mathrm{d\lambda}}{d\tau_{2}}+J_{1}\frac{\mathrm{d\lambda}}{d\tau_{2}}+\left(J_{2}\frac{\mathrm{d\lambda}}{d\tau_{2}}e^{-\lambda \tau_{2}}+\left(J_{2}\lambda +k_{2}\right)\frac{d}{d\tau_{2}}\left(e^{-\lambda \tau_{2}}\right)\right)=0$$ (12)

$$\frac{d}{d\tau_{2}}\left(e^{-\lambda \tau_{2}}\right)=-\lambda e^{-\lambda \tau_{2}}-\tau_{2}\frac{\mathrm{d\lambda}}{d\tau_{2}}e^{-\lambda \tau_{2}}$$

$$2\lambda \frac{\mathrm{d\lambda}}{d\tau_{2}}+J_{1}\frac{\mathrm{d\lambda}}{d\tau_{2}}+J_{2}\frac{\mathrm{d\lambda}}{d\tau_{2}}e^{-\lambda \tau_{2}}+\left(J_{2}\lambda +k_{2}\right)\left(-\lambda e^{-\lambda \tau_{2}}-\tau_{2}\frac{\mathrm{d\lambda}}{d\tau_{2}}e^{-\lambda \tau_{2}}\right)=0$$

$$2\lambda \frac{\mathrm{d\lambda}}{d\tau_{2}}+J_{1}\frac{\mathrm{d\lambda}}{d\tau_{2}}+J_{2}\frac{\mathrm{d\lambda}}{d\tau_{2}}e^{-\lambda \tau_{2}}-\left(J_{2}\lambda +k_{2}\right)\lambda e^{-\lambda \tau_{2}}-\left(J_{2}\lambda +k_{2}\right)\tau_{2}\frac{\mathrm{d\lambda}}{d\tau_{2}}e^{-\lambda \tau_{2}}=0$$

$$\left(2\lambda +J_{1}+J_{2}e^{-\lambda \tau_{2}}-\left(J_{2}\lambda +k_{2}\right)\tau_{2}e^{-\lambda \tau_{2}}\right)\frac{\mathrm{d\lambda}}{d\tau_{2}}=\left(J_{2}\lambda +k_{2}\right)\lambda e^{-\lambda \tau_{2}}$$

$$\frac{\mathrm{d\lambda}}{d\tau_{2}}=\frac{\left(J_{2}\lambda +k_{2}\right)\lambda e^{-\lambda \tau_{2}}}{2\lambda +J_{1}+J_{2}e^{-\lambda \tau_{2}}-\left(J_{2}\lambda +k_{2}\right)\tau_{2}e^{-\lambda \tau_{2}}}$$

$${\left[\frac{\mathrm{d\lambda}}{d\tau_{2}}\right]}^{-1}=\frac{d\tau_{2}}{\mathrm{d\lambda}}=\frac{2\lambda +J_{1}+J_{2}e^{-\lambda \tau_{2}}-\left(J_{2}\lambda +k_{2}\right)\tau_{2}e^{-\lambda \tau_{2}}}{\left(J_{2}\lambda +k_{2}\right)\lambda e^{-\lambda \tau_{2}}}$$

Recall that:

$$\begin{array}{c} \lambda^{2}+J_{1}\lambda +k_{1}+\left(J_{2}\lambda +k_{2}\right)e^{-\lambda \tau_{2}}\\ -\lambda^{2}+J_{1}\lambda +k_{1}=\left(J_{2}\lambda +k_{2}\right)e^{-\lambda \tau_{2}} \end{array}$$

Replacing this in the equation.

2λ+J1−λ(λ2+J1λ+k1)+J2λ(J2λ+k2)−τ2λddτ2(Re(λ))−1=Re(dλdτ2)−1|λ=iρ=1w(2w(w2−k0)+J1w(w2−k0)2+(J1w)2−J1w(J1w)2+k22)(w2−k1)+(J1w2)2=(J2w)2+k22

Given the condition $J_{1}^{2}+J_{2}^{2}-2k_{1}$ >0 , it follows that:

ddτRe(λ)−1|λ=iw>0,

which ensures that the transversality condition is satisfied. This, in turn, leads to the occurrence of a Hopf bifurcation at ω=w and $\tau_{2}=\tau \left(0\right)$ . As a result, the equilibrium $P^{*}$ of system (5) is asymptotically stable for $\tau_{2}\in (0,\tau \left(0\right)$ , and undergoes a Hopf bifurcation at $\tau_{2}=\tau \left(0\right)$ , marking a significant change in the system’s dynamical behavior.

## 6. Global asymptotically stability

From equation (5), we have

$$\frac{\mathrm{dS}}{\mathrm{dt}}=\Lambda -\mathrm{\mu S}-\frac{\mathrm{\beta S}\left(t-\tau_{2}\right)I\left(t-\tau_{2}\right)e^{-\mu \tau_{2}}}{1+\mathrm{mI}\left(t-\tau_{2}\right)}$$

$$\frac{\mathrm{dI}}{\mathrm{dt}}=\frac{\mathrm{\beta S}\left(t-\tau_{2}\right)I\left(t-\tau_{2}\right)e^{-\mu \tau_{2}}}{1+\mathrm{mI}\left(t-\tau_{2}\right)}-\rho R_{1}+\left(\mu +\delta +\mathrm{c\tau}+\left(1-c\right)\tau \right)I$$

can be re-written as

$$\frac{\mathrm{dS}}{\mathrm{dt}}=\Lambda -\mathrm{\mu S}-U\left(S\left(t\right),I\left(t\right)\right)$$

$$\frac{\mathrm{dI}}{\mathrm{dt}}=U\left(S\left(t\right),I\left(t\right)\right)+V\left(I\left(t\right)\right)$$

The Lyapunov direct method, as formulated by Huang et al, ^41^ provides a systematic approach for establishing the global stability of equilibria in dynamical systems. This method involves the construction of a suitable scalar-valued function, known as a Lyapunov function, which satisfies specific positivity and derivative conditions along system trajectories. In this section, we construct an appropriate Lyapunov functional adapted to the structure of the model (5) and employ it to thoroughly analyze the global stability of the system’s equilibrium points.

The incidence function U(S(t),I(t)) is expressed as the product U(S(t))W(I(t)) .

V(S(t))=βS(t)

$$W\left(I\left(t\right)\right)=\frac{I\left(t\right)}{1+\mathrm{mI}\left(t\right)}$$

$$Y\left(I\left(t\right)\right)=\rho R_{1}+\mathrm{zI},\;\text{where}\;z=\left(\mu +\delta +\mathrm{c\tau}\left(1-c\right)\tau \right)$$

and impose the following assumptions:

1. The functions U and W satisfy U(0)=W(0)=0 and their first derivatives $V^{\prime }\left(S\right)$ and $W^{\prime }\left(I\right)$ are strictly positive for all S,I>0 .
2. Either $\frac{W\left(I\left(0\right)\right)}{W\left(I^{*}\right)}\;\geq \;1$ or $\frac{Y\left(I\left(t\right)\right)}{V\left(S\left(t\right)\right)W\left(I\left(t\right)\right)}\;\leq \;1$ for all S,I≥0 .
3. The function W is concave in I , i.e., $W^{\prime }\left(I\right)>0$ and $\frac{\partial^{2}W\left(I\right)}{\partial I^{2}}$ for all S,I>0 .

On this premise, we state the following theorem:

*5. Theorem*

1. Under the conditions 1 and 2, the equilibrium point $P^{*}\left(S^{*},I^{*}\right)$ is globally stable for any delay parameter $\tau_{2}\;\geq \;0$ .
2. If conditions (1)–(3) hold and $R_{0}<1$ , the DFE is stable for $\tau_{2}\;\geq \;0$ .

Proof If we define

$$F_{1}\left(S\left(t\right),I\left(t\right)\right)=S\left(t\right)-V\left(S\left(t\right)\right)\int_{S^{*}}^{S\left(t\right)}\frac{\mathrm{d\phi}}{V\left(\phi \right)}+I\left(t\right)-W\left(I\left(t\right)\right)\int_{I^{*}}^{I\left(t\right)}\frac{\mathrm{d\phi}}{W\left(\phi \right)}$$

Differentiating by Leibniz’s rule,

$$\frac{\partial F_{1}}{\partial S}=1-\frac{V\left(S^{*}\right)}{V\left(S\right)}$$

$$\frac{\partial F_{1}}{\partial I}=1-\frac{W\left(I^{*}\right)}{W\left(I\right)}$$

Since

$$\frac{\partial^{2}F_{1}}{\partial S^{2}}=\frac{V\left(S\right)U\left(S^{*}\right)}{{\left(V\left(S\right)\right)}^{2}}>0\;\text{and}$$

$$\frac{\partial^{2}F_{1}}{\partial I^{2}}=\frac{W\left(I\right)U\left(I^{*}\right)}{{\left(W\left(I\right)\right)}^{2}}>0$$

$$\frac{\partial^{2}F_{1}}{\partial S\partial I}=0$$

Thus, $P^{*}\left(S^{*},I^{*}\right)$ is globally minimum.

Taking the total derivatives

$$\frac{dF_{1}}{\mathrm{dt}}=\frac{\mathrm{dS}}{\mathrm{dt}}\left[1-\frac{V\left(S^{*}\right)}{V\left(S\right)}\right]+\frac{\mathrm{dI}}{\mathrm{dt}}\left[1-\frac{W\left(I^{*}\right)}{W\left(I\right)}\right]$$

$$\frac{dF_{1}}{\mathrm{dt}}=\left[V\left(S^{*}\right)W\left(I^{*}\right)-\mu S^{*}-\frac{V\left(S\left(t-\tau_{2}\right)W\left(I\left(t-\tau_{2}\right)\right)\right)}{1+\mathrm{mI}\left(t-\tau_{2}\right)}\right]$$

$$\times \left[1-\frac{V\left(S,x^{*}\right)}{V\left(S\right)}\right]+\left[\frac{V\left(S\left(t-\tau_{2}\right)\right)W\left(t-\tau_{2}\right)}{1+\mathrm{WI}\left(t-2\phi \right)}+\rho R_{1}+\mathrm{ZI}\right]\left[1-\frac{W\left(I^{*}\right)}{W\left(I\right)}\right]$$

Let’s assume

For $F_{2}$ :

$$\begin{array}{c} F_{2}=\int_{0}^{\tau_{2}}\frac{W(I\left(t-\phi \right))}{W\left(t^{*}\right)}-1-\ln\left[\frac{W\left(I\left(t-\phi \right)\right)}{W\left(t\right)}\right]\mathrm{d\phi}\\ \frac{dF_{2}}{\mathrm{dt}}=\frac{d}{\mathrm{dt}}\int_{0}^{\tau_{2}}\frac{W\left(I\left(t-\phi \right)\right)}{W\left(I\left(t^{*}\right)\right)}-1-\ln\left[\frac{W\left(I\left(t-\phi \right)\right)}{W\left(I\left(t\right)\right)}\right]\mathrm{d\phi}\\ =-\int_{0}^{\tau_{2}}\frac{d}{\mathrm{d\phi}}\left[\frac{W\left(I\left(t\int \phi \right)\right)}{W\left(I^{*}\right)}\int 1-\ln\left(\frac{W\left(I\left(t-\phi \right)\right)}{W\left(I\left(t\right)\right)}\right)\right]\mathrm{d\phi}\\ -\frac{W\left(t-\tau_{2}\right)}{W\left(I\right)}+\frac{W(I\left(t)\right)}{W\left(I^{*}\right)}+\ln\left(\frac{W\left(I\left(t-\tau_{2}\right)\right)}{W\left(I\left(t\right)\right)}\right) \end{array}$$

Let’s define the Lyapunov function

$$F=F_{1}+GF_{2}\;\text{where}\;G=V\left(S^{*}\right)W\left(I^{*}\right)$$

$$\frac{\mathrm{dF}}{\mathrm{dt}}=\frac{dF_{1}^{*}}{\mathrm{dt}}+G\frac{dF_{2}}{\mathrm{dt}}$$

$$\begin{array}{c} \frac{\mathrm{dF}}{\mathrm{dt}}=\left[V\left(S^{*}\right)W\left(I^{*}\right)-\mu S^{*}-\frac{V\left(S\left(t-\tau_{2}\right)\right)W\left(I\left(t-\tau_{2}\right)\right))}{1+\mathrm{mI}\left(t-\tau_{2}\right)}\right]\left[1-\frac{V\left(S^{*}\right)}{V\left(S\right)}\right]\\ +\left[\frac{V(S\left(\left(t-\tau_{2}\right)W\left(t-\tau_{2})\right)\right)}{1+\mathrm{mI}\left(t-\tau_{2}\right)}+y(I\left(t)\right)\right]\left[\frac{1-W\left(I\left(t\right)\right)}{W\left(I\left(t\right)\right)}\right]\\ +V\left(S^{*}\right)W\left(T^{*}\right)\left[\frac{-W(I\left(t-\tau_{2})\right)}{W\left(I^{*}\right)}+\frac{W(I\left(T)\right)}{W\left(I^{*}\right)}+\ln\frac{\mathrm{WI}\left(t-\tau_{2}\right)}{\mathrm{WI}\left(t\right)}\right] \end{array}$$

Let’s use;

$$\begin{array}{c} \ln\frac{W\left(I\left(t-\tau_{2}\right)\right)}{W\left(I\left(t\right)\right)}=\ln\left(\frac{V\left(S^{*}\left(1+\ln\left(t-\tau_{2}\right)\right)\right)}{V\left(S\left(t-\tau_{2}\right)\right)}\right)+\ln\left[\frac{V(S\left(t-\tau_{2}\right)W\left(I\left(t-\tau_{2}\right)\right)}{W(I\left(t\right)V\left(S^{*}\left(1+\ln\left(t-\tau_{2}\right)\right)\right)}\right]\\ \begin{array}{c} \frac{\mathrm{dF}}{\mathrm{dt}}=\left[1-\frac{V\left(S*\right)}{V\left(S\right)}+\ln\frac{V\left(S^{*}\right)\left(1+\mathrm{mI}\left(t-\tau_{2}\right)\right)}{V\left(s\left(t-\tau_{2}\right)\right)}\right]V\left(S^{*}\right)W\left(I^{*}\right)\\ +\left[V\left(S^{*}\right)-V\left(S\right))\right]\left[\mu S^{*}+\frac{V\left(S\left(t-\tau_{2}\right)\right)W\left(I\left(t-\tau_{2}\right)\right)}{1+\mathrm{mI}\left(t-\tau_{2}\right)}\right]\frac{1}{V\left(S\right)}\\ \begin{array}{c} +\left[V\left(S^{*}\right)W\left(I^{*}\right)\right]\left[1-\frac{V\left(S\left(t-\tau_{2}\right)\right)W(I\left(t-\tau_{2}\right)}{V\left(S^{*}\right)\left(1+\mathrm{mI}\left(t-\tau_{2}\right)\right)}\right]+\ln\left[\frac{V\left(S\left(t-\tau_{2}\right)\right)W\left(I\left(t-\tau_{2}\right)\right)}{W\left(I\left(t\right)V\left(S^{*}\right)\right)\left(1+\mathrm{mI}\left(t-\tau_{2}\right)\right)}\right]\\ +V\left(S^{*}\right)W\left(I^{*}\right)\left[1-\frac{W\left(I\left(t\right)\right)}{W\left(I^{*}\right)}\right]\left[1-\frac{W\left(I^{*}\right)}{W\left(I\right)}\right] \end{array} \end{array} \end{array}$$

If we have

$$[1-\frac{V\left(S^{*}\right)}{V\left(S\right)}+\ln\left(\frac{V\left(S^{*}\right)\left(1+\mathrm{mI}\left(t-\tau_{2}\right)\right)}{V(S\left(t-\tau_{2}\right)}\right))]<0$$

and

$$\left[1-\frac{V\left(S\left(t-\tau_{2}\right)\right)W\left(I\left(t-\tau_{2}\right)\right)}{V\left(S^{*}\right)\left(1+\mathrm{mI}\left(t-\tau_{2}\right)\right)}+\mu \frac{V\left(S\left(t-\tau_{2}\right)\right)W\left(I\left(t-\tau_{2}\right)\right)}{W\left(I\left(t\right)\right)V\left(S^{*}\right)\left(1+\mathrm{mI}\left(t-\tau_{2}\right)\right)}\right]<0$$

Also

$$V\left(S\right)>V\left(S^{*}\right)\;\forall S\left(t\right)>S^{*},$$

hence

$$\left[V\left(S^{*}\right)-V\left(S\right)\right]\left[\mu S^{*}+\frac{V\left(S\left(t-\tau_{2}\right)\right)W\left(I\left(t-\tau_{2}\right)\right)}{1+\mathrm{mI}\left(t-\tau_{2}\right)}\right]\;\leq \;0$$

Hence we can say that,

$$\frac{\mathrm{dF}}{\mathrm{dt}}\;\leq \;0,$$

This is sufficient for Corollary 5.2 of [Kuang (1993). ^31^ We can then say that the endemic state $P^{*}\left(S^{*},I^{*}\right)$ is globally asymptotically stable.

## 7. Discussion of Results

**Figure 2.**
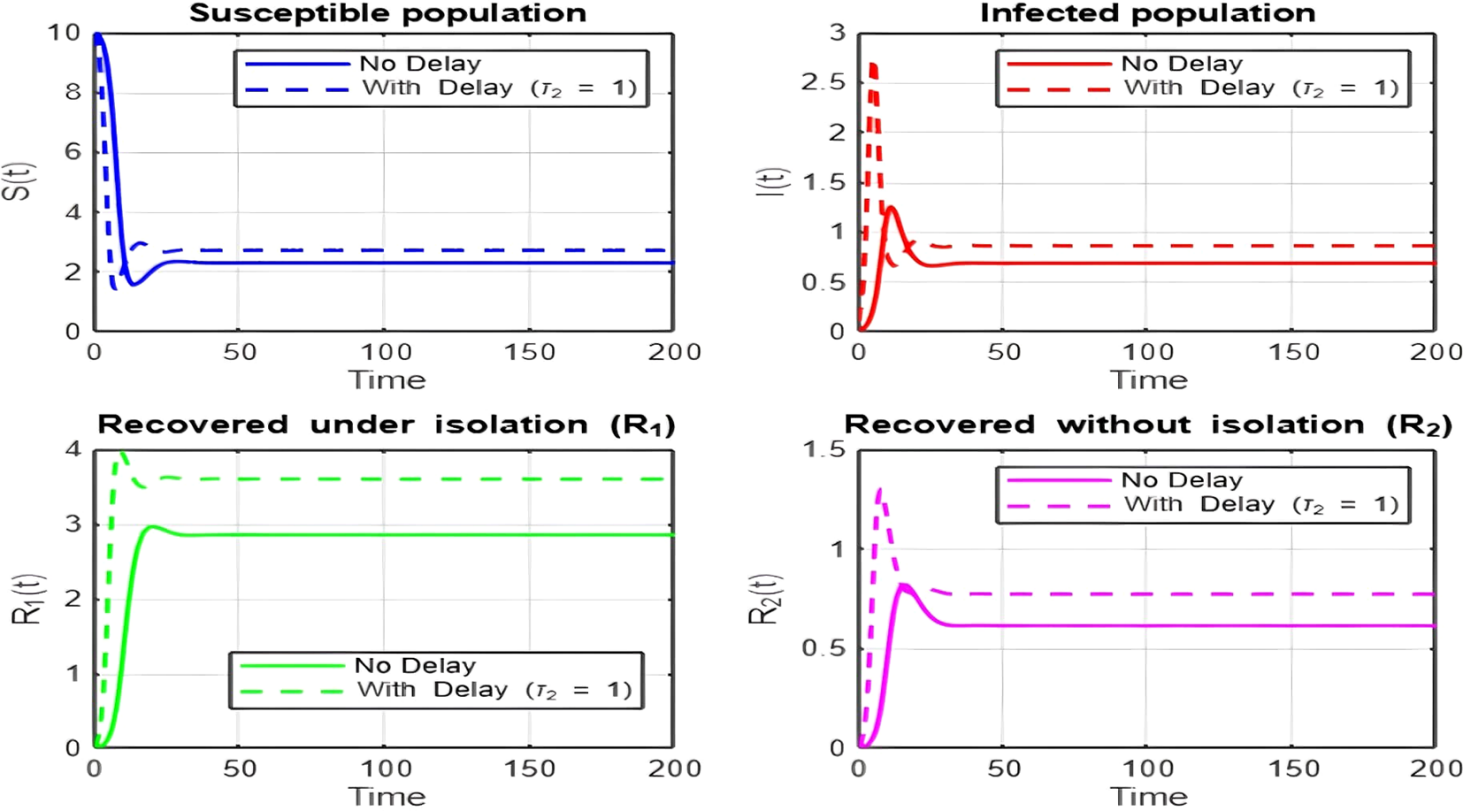
Time series (with and without delay) for tau2 = 1; lambda = 1; mu = 0.1; beta = 0.5; mu2 = 0.1; m = 0.5; p = 0.05; delta = 0.2; c = 0.7; rho = 0.1; tau = 0.6; theta = 0.3; S0 = 10; I0 = 0.1; R10 = 0; R20 = 0; tspan = [0, 200].

**Figure 3.**
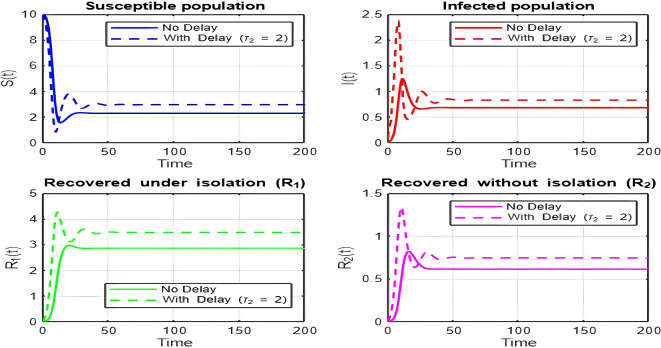
Time series (with and without delay) for tau2 = 2; lambda = 1; mu = 0.1; beta = 0.5; mu2 = 0.1; m = 0.5; p = 0.05; delta = 0.2; c = 0.7; rho = 0.1; tau = 0.6; theta = 0.3; S0 = 10; I0 = 0.1; R10 = 0; R20 = 0; tspan = [0, 200].

**Figure 4.**
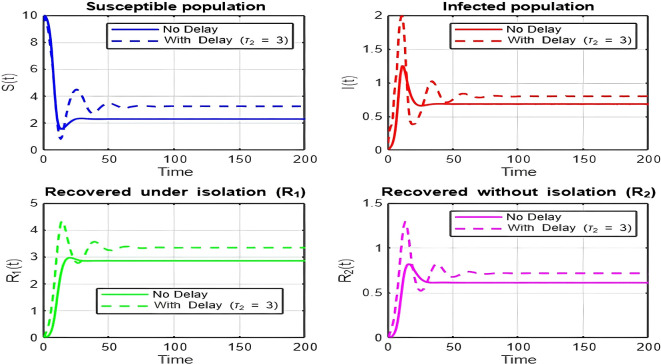
Time series (with and without delay) for tau2 = 3; lambda = 1; mu = 0.1; beta = 0.5; mu2 = 0.1; m = 0.5; p = 0.05; delta = 0.2; c = 0.7; rho = 0.1; tau = 0.6; theta = 0.3; S0 = 10; I0 = 0.1; R10 = 0; R20 = 0; tspan = [0, 200].

**Figure 5.**
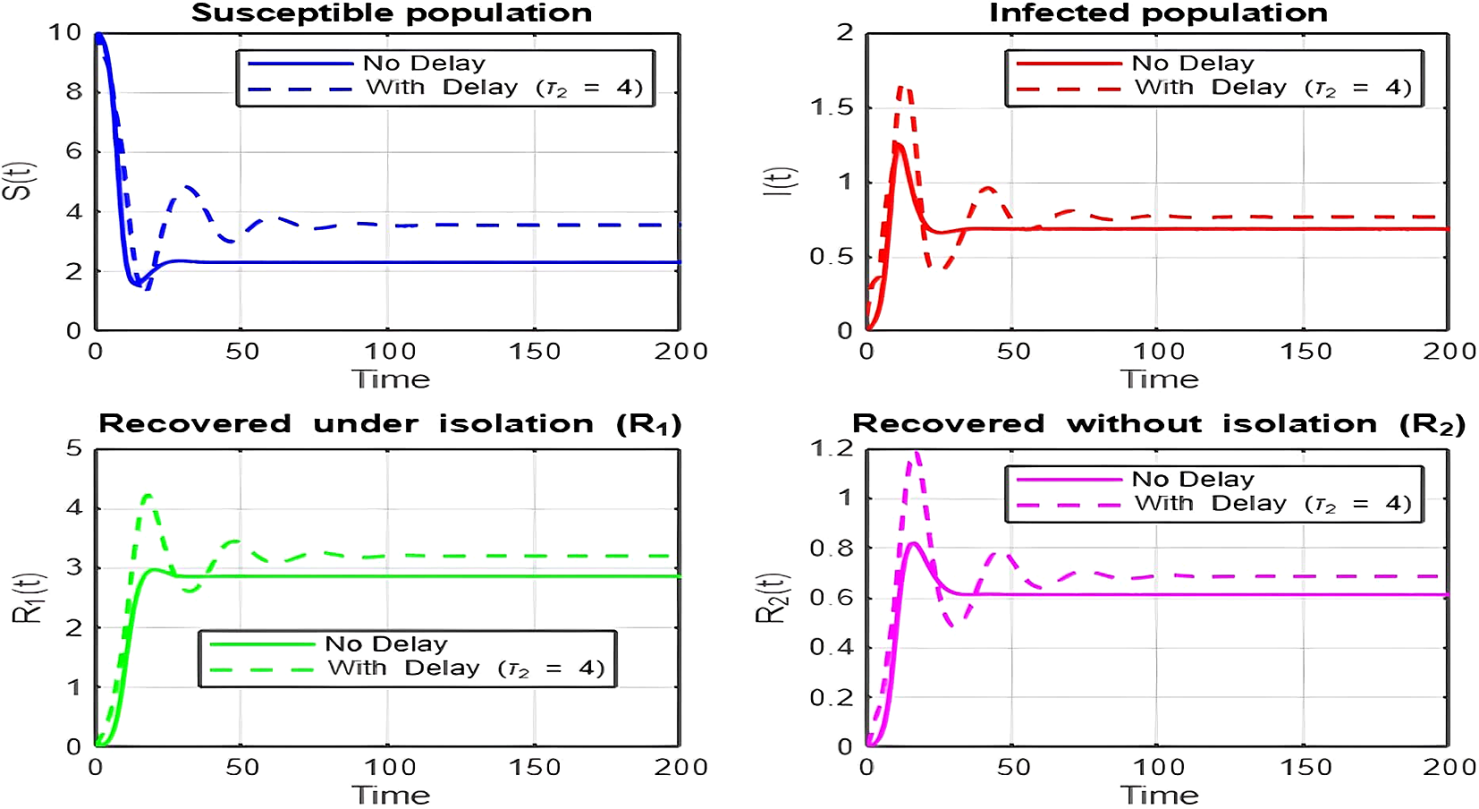
Time series (with and without delay) for tau2 = 4; lambda = 1; mu = 0.1; beta = 0.5; mu2 = 0.1; m = 0.5; p = 0.05; delta = 0.2; c = 0.7; rho = 0.1; tau = 0.6; theta = 0.3; S0 = 10; I0 = 0.1; R10 = 0; R20 = 0; tspan = [0, 200].

**Figure 6.**
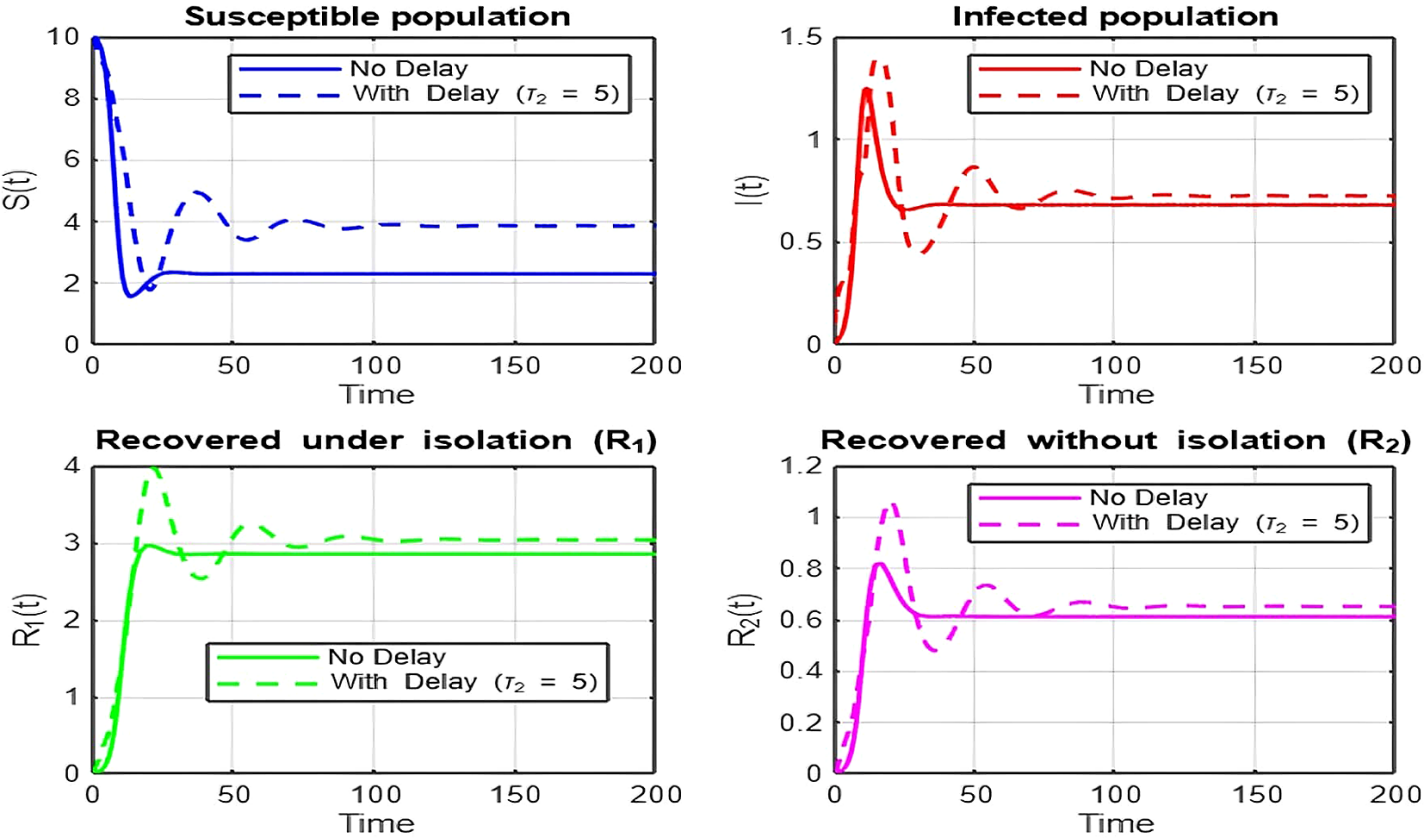
Time series (with and without delay) for tau2 = 5; lambda = 1; mu = 0.1; beta = 0.5; mu2 = 0.1; m = 0.5; p = 0.05; delta = 0.2; c = 0.7; rho = 0.1; tau = 0.6; theta = 0.3; S0 = 10; I0 = 0.1; R10 = 0; R20 = 0; tspan = [0, 200].

**Figure 7.**
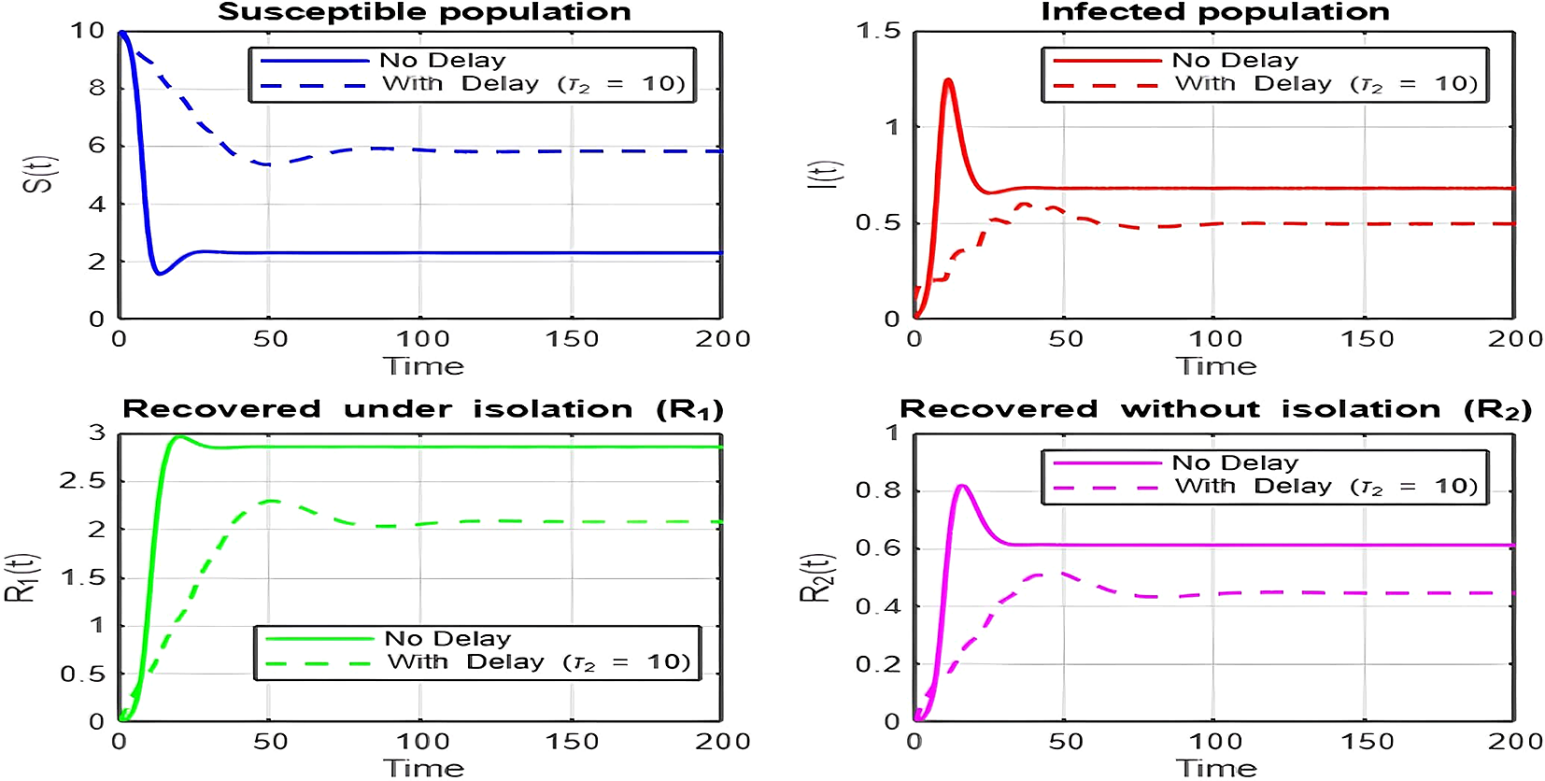
Time series (with and without delay) for tau2 = 10; lambda = 1; mu = 0.1; beta = 0.5; mu2 = 0.1; m = 0.5; p = 0.05; delta = 0.2; c = 0.7; rho = 0.1; tau = 0.6; theta = 0.3; S0 = 10; I0 = 0.1; R10 = 0; R20 = 0; tspan = [0, 200].

**Figure 8.**
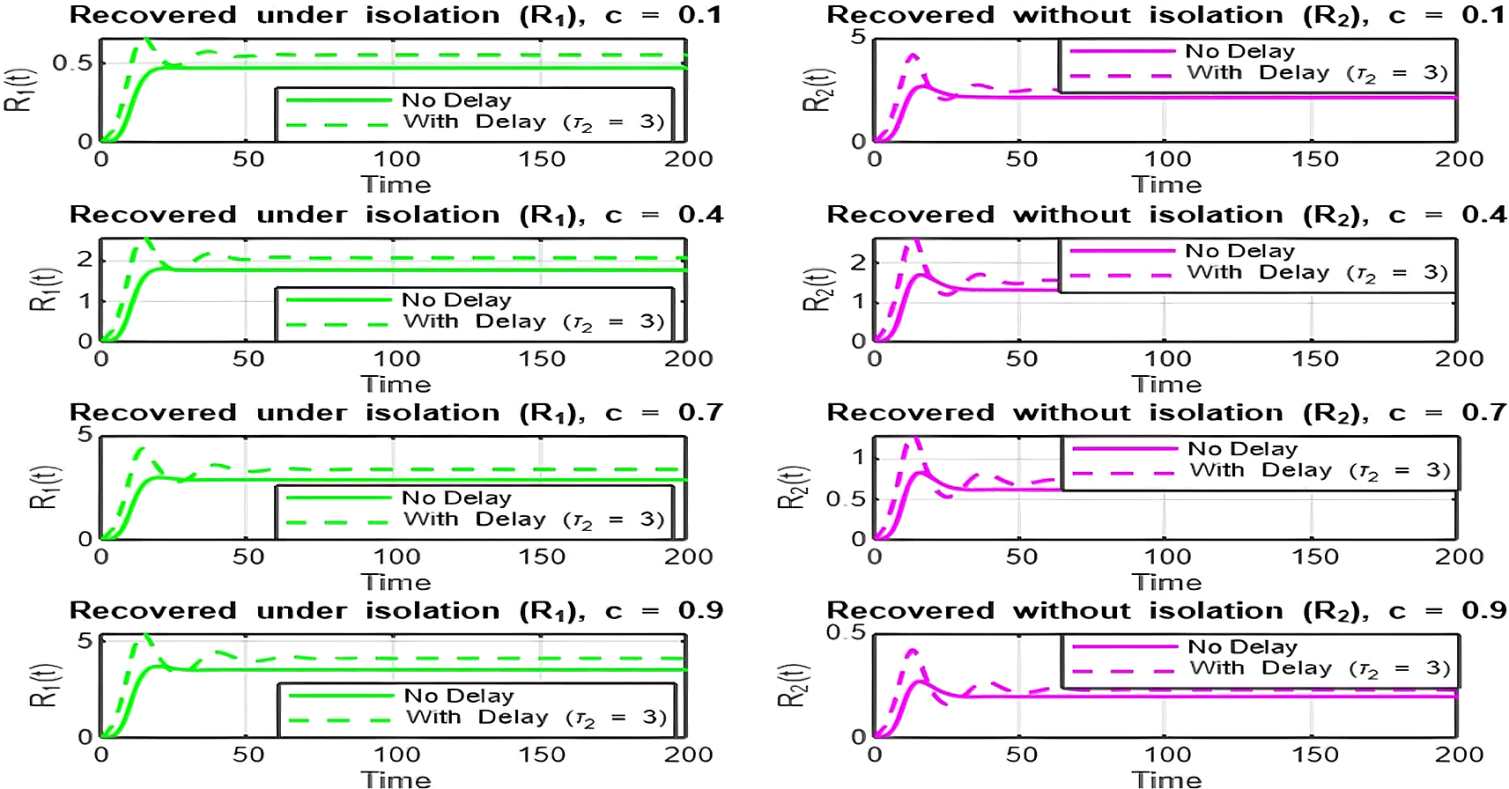
Time series (with and without delay) for both R_1 and R_2 where tau2 = 3; lambda = 1; mu = 0.1; beta = 0.5; mu2 = 0.1; m = 0.5; p = 0.05; delta = 0.2; c = 0.7; rho = 0.1; tau = 0.6; theta = 0.3; S0 = 10; I0 = 0.1; R10 = 0; R20 = 0; tspan = [0, 200].

1. Figures (2)- (8) **and** Figure 17 **: Dynamics Comparison (With and Without Delay) [** Figure 2, Figure 3, Figure 4, Figure 5, Figure 6, Figure 7 ***,*** Figure 8. **]**
  The dynamics comparison plot demonstrates the effect of incorporating a delay ( $\tau_{2}$ ) in the recovery process on the population dynamics of susceptible ( S ) and infected ( I ) individuals.
  • **Without Delay:**
    The system without delay ( $\tau_{2}=0$ ) exhibits a faster approach to equilibrium, reflecting a more straightforward trajectory to either disease-free equilibrium (DFE) or endemic equilibrium (EE), depending on parameter values. This is consistent with classical models, where recovery and transitions between compartments occur instantaneously or without delays.
  • **With Delay:**
    Introducing a delay ( $\tau_{2}>0$ ) introduces oscillatory behavior in the infected population due to the feedback loop created by the delayed transitions. These oscillations may represent cycles of outbreaks and remissions, commonly observed in zoonotic diseases like Ebola, where external factors such as virus persistence in bodily fluids or immune system delays affect recovery rates.
    The presence of delay slows down convergence to equilibrium and may indicate higher disease prevalence over time. These dynamics underscore the critical role of delayed feedback in influencing outbreak persistence and stability in real-world scenarios.
2. **Figure 12 and Figures 13– Figure 15: Phase Space (Trajectories in** $S,I,R_{1}$ **and** $S,I,R_{2}$ **)**
  The phase space plot provides insights into the long-term behavior of the model with and without delay.
  • **Disease-Free Equilibrium (DFE):**
    The DFE point (marked as black in the plot) corresponds to the scenario where the infection dies out completely ( I=0 , $R_{1}=R_{2}=0$ ). This equilibrium is stable for small transmission rates ( β ) or when the basic reproduction number ( $R_{0}$ ) is less than 1.
  • **Endemic Equilibrium (EE):**
    The endemic equilibrium (marked as green) represents a state where the infection persists in the population. This occurs when $R_{0}>1$ , allowing for a sustained infected population. For the delayed system, trajectories often show oscillations before converging to the EE, reflecting periodic outbreaks.
    The trajectory with delay exhibits more complex behavior (such as spiraling orbits) before stabilization, highlighting the destabilizing impact of delayed recovery on the population dynamics.
3. Figure 16: Bifurcation Analysis (Peak Infection vs. Delay $\tau_{2}$ )
  The bifurcation diagram investigates how varying the delay ( $\tau_{2}$ ) influences peak infection levels.
  • **Without Delay (** $\tau_{2}=0$ **):**
    Peak infection levels remain relatively constant as the system lacks the feedback loop induced by delay. The dynamics stabilize quickly at the endemic equilibrium.
  • **With Delay (** $\tau_{2}>0$ **):**
    Introducing delay leads to increasing peak infection levels as $\tau_{2}$ grows. This is a direct consequence of the prolonged infectious period caused by delays in transitions to recovery ( $R_{1}$ and $R_{2}$ ). Moreover, as $\tau_{2}$ increases beyond a critical threshold, the system exhibits oscillatory outbreaks, as seen in the dynamics plot.
    The bifurcation plot effectively distinguishes between the disease-free region ( $R_{0}<1$ ) and the endemic region ( $R_{0}>1$ ) by showing when peak infection levels transition from zero to non-zero values. The critical value of $\tau_{2}$ at which this transition occurs aligns with thresholds derived from the model’s reproduction number.
4. **Isolation Parameter** c

**Figure 9.**
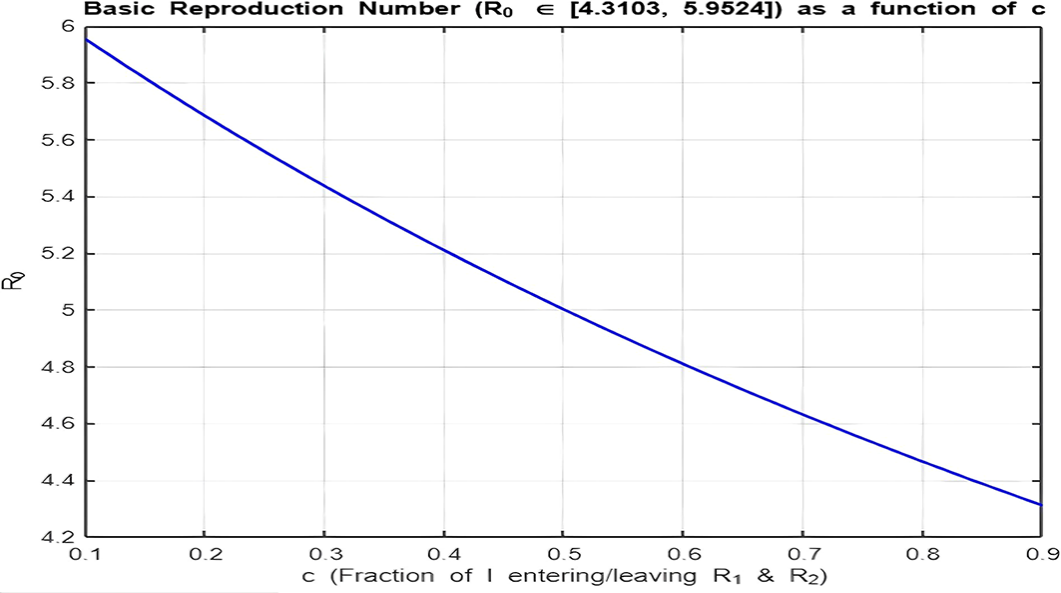
Relationship between basic reproduction number R_0 and the isolation parameter c for R_0 > 1.

**Figure 10.**
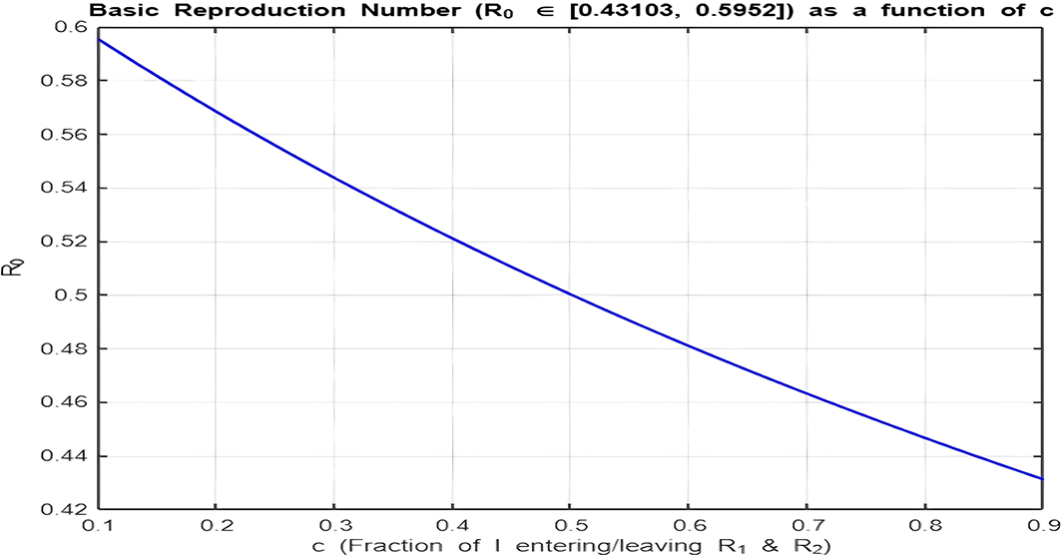
Relationship between basic reproduction number R_0 and the isolation parameter c for R_0 < 1.

**Figure 11.**
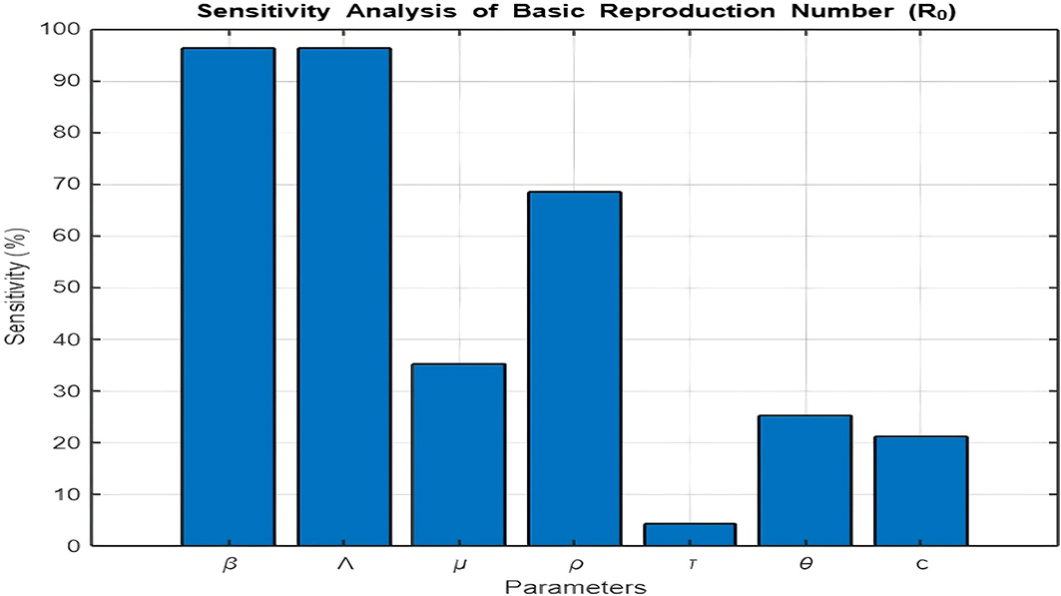
How each parameter contribute to the basic reproduction number.

  The isolation parameter c reflects the fraction of recovered individuals ( $R_{1}$ ) who effectively isolate themselves and avoid further transmission. The two plots ( Figure 9 and Figure 10) show how $R_{0}$ depends on c , and the behavior is analyzed for $R_{0}>1$ and $R_{0}<1$ :
  • Figure 9 **(** $R_{0}>1$ **):**
    With the range [4.3,5.9] , there is a decreasing relationship between c and $R_{0}$ , indicating that as the isolation parameter c increases, $R_{0}$ decreases. Increasing c reduces the effective transmission by isolating recovered individuals, helping to lower $R_{0}$ .
  • Figure 10. **(** $R_{0}<1$ **):**
    With the range [0.43,0.59] , there is a decreasing relationship between c and $R_{0}$ , but $R_{0}$ values are already below 1. Increasing c further accelerates the decline, making it harder for the infection to persist in the population. Here, lower isolation efforts (small c ) are sufficient to maintain $R_{0}<1$ .
5. Figure 11 **: Sensitivity Analysis of** $R_{0}$

Figure 11 presents the sensitivity analysis of the basic reproduction number ( $R_{0}$ ) with respect to model parameters. The results reveal that the transmission rate ( β ), recruitment rate ( Λ ), and recovery rate into $R_{2}$ ( ρ ) are the parameters that contribute most significantly to $R_{0}$ .

**Table 1.** Parameters of the SEIRR Ebola transmission model.

Parameter	Description
Λ	Recruitment rate of susceptible individuals
μ	Natural mortality rate
β	Disease transmission rate
θ	Rate at which exposed individuals become infectious
δ	Ebola-induced mortality rate
c	Proportion of cases that recover in isolation
τ	Total recovery rate (sum of isolated and non-isolated recovery)
ρ	Rate at which non-isolated recovered individuals transition back to infectious status due to viral persistence, enabling potential transmission to susceptible individuals

**Figure 12.**
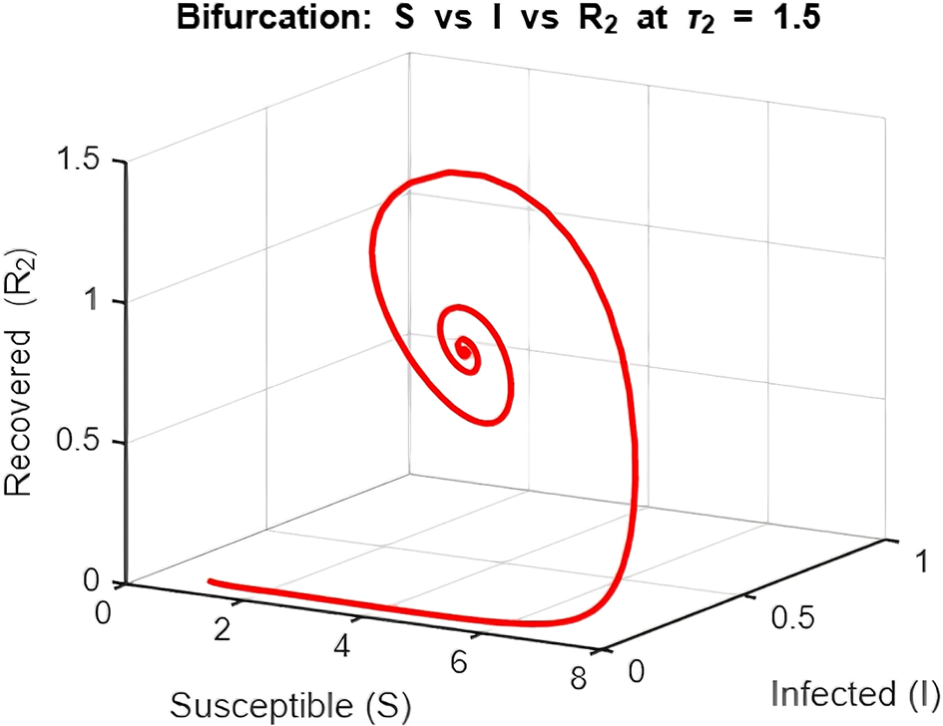
Phase space of equilibrium E* ( S*, *, R*) when the delay is 1.5 i.e tau2 = 1.5. for lambda = 1; mu = 0.1; beta = 0.5; mu2 = 0.1; m = 0.05; delta = 0.2; c = 0.7; rho = 0.1; p = 0.05; S0 = 10; I0 = 0.1; R10 = 0; R20 = 0; tspan = [0, 200].

**Figure 13.**
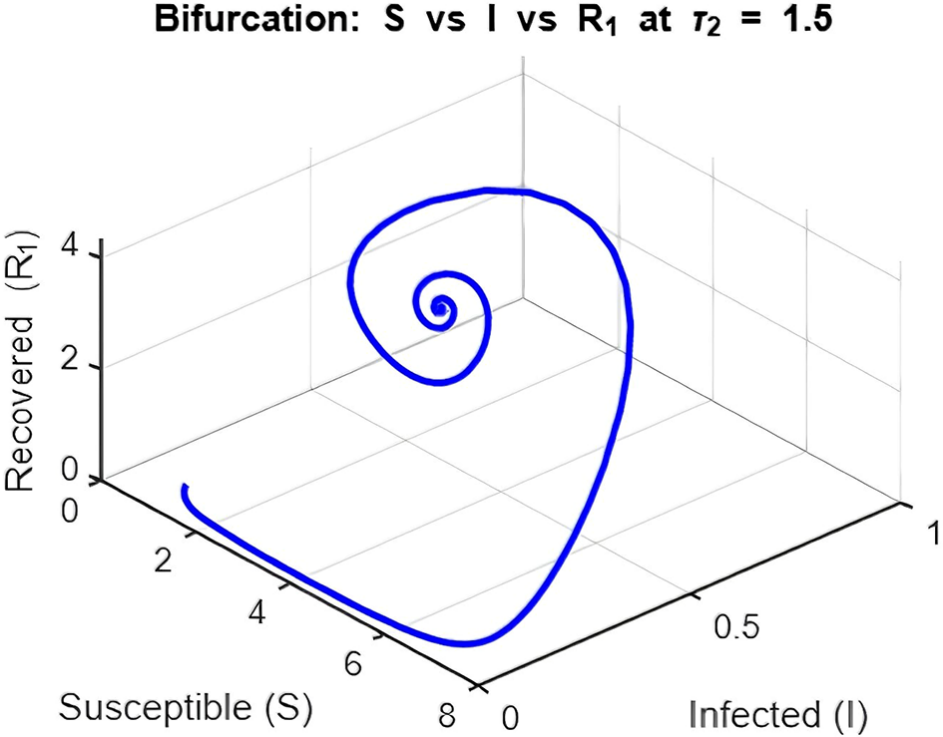
EE = [2.2313 1.3254 5.5867], DFE = [13 0 0]. Lambda = 1.30; mu = 0.1; beta = 0.5; delta = 0.2; rho = 0.1; c = 0.7; tau = 0.6; tau2 = 2.5; m = 0.05; mu2 = 0.1; S0 = 5; I0 = 1; R10 = 0; R20 = 0; tspan = [0, 300].

**Figure 14.**
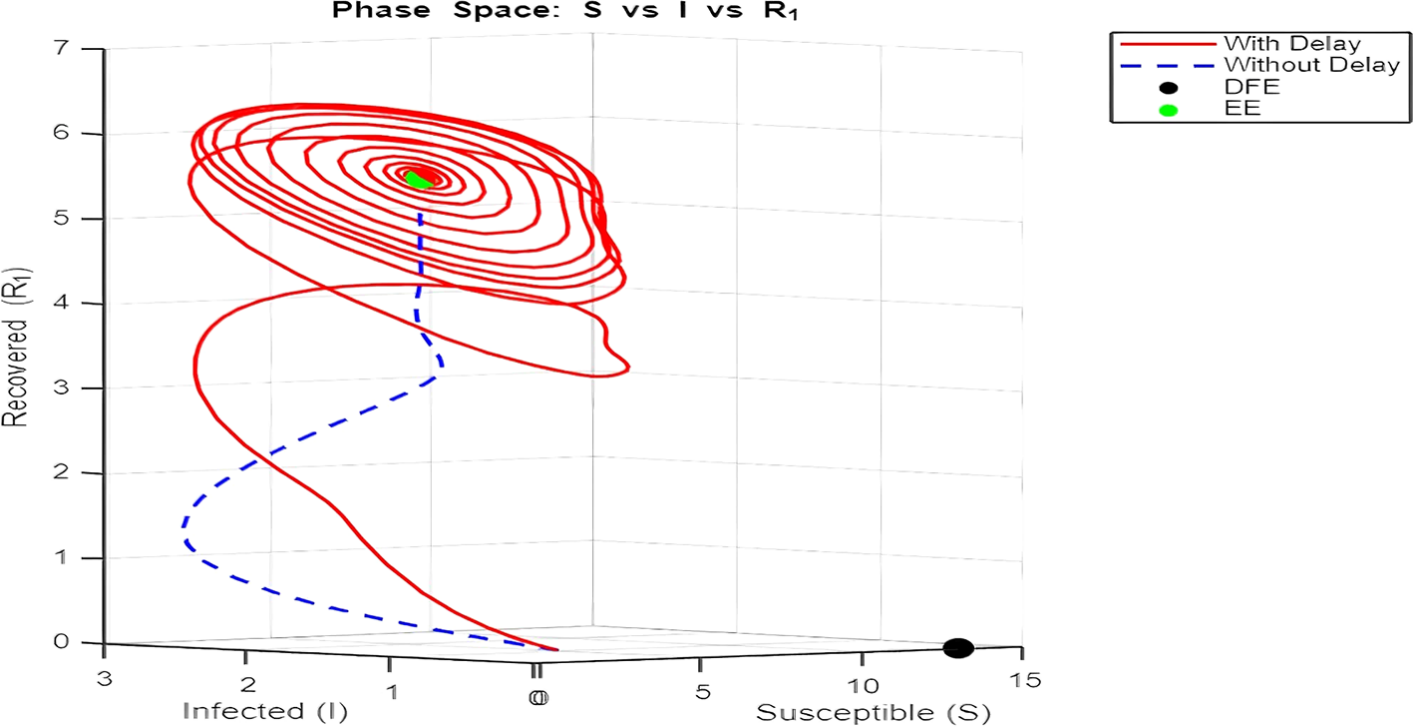
EE = [2.2313 1.3254 1.1963], DFE = [13 0 0]. Lambda = 1.30; mu = 0.1; beta = 0.5; delta = 0.2; rho = 0.1; c = 0.7; tau = 0.6; tau2 = 2.5; m = 0.05; mu2 = 0.1; S0 = 5; I0 = 1; R10 = 0; R20 = 0; tspan = [0, 300].

**Figure 15.**
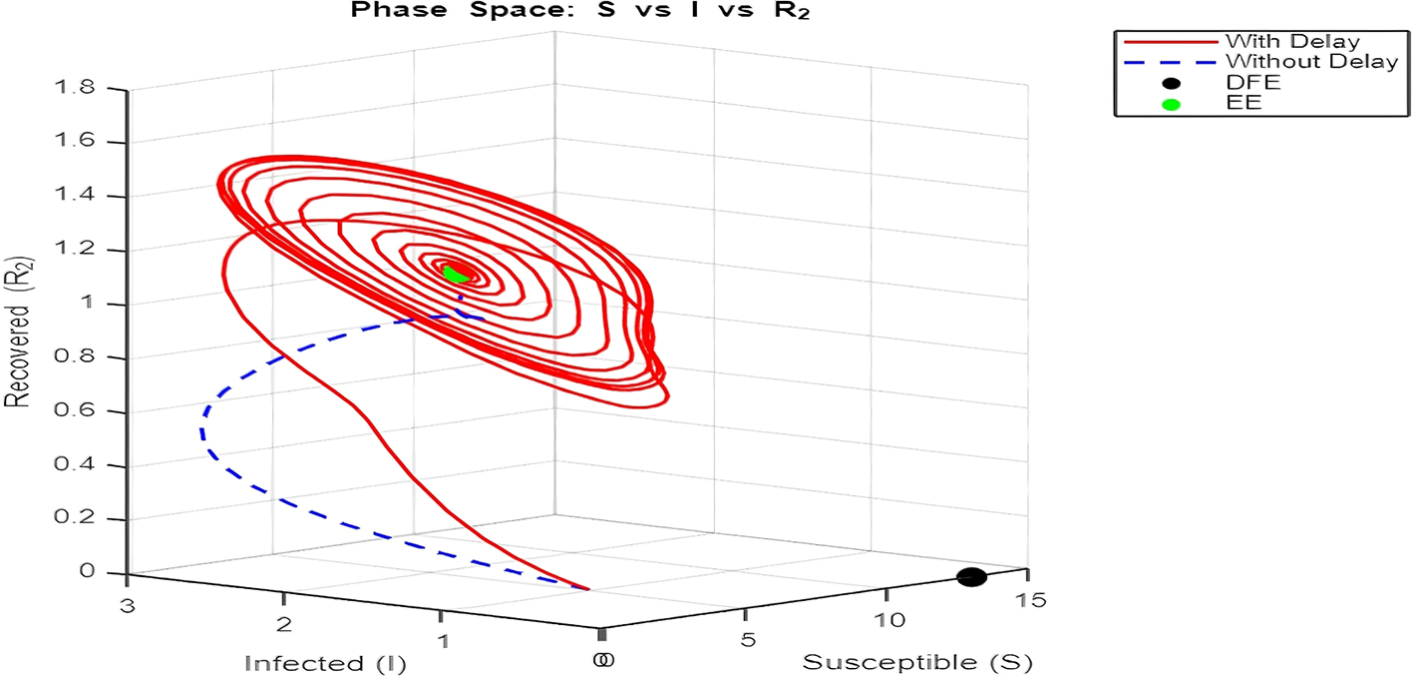
Lambda = 1.30; mu = 0.1; beta = 0.5; delta = 0.2; rho = 0.1; c = 0.7; tau = 0.6; tau2 = 2.5; m = 0.05; mu2 = 0.1; S0 = 5; I0 = 1; R10 = 0; R20 = 0; tspan = [0, 300].

**Figure 16.**
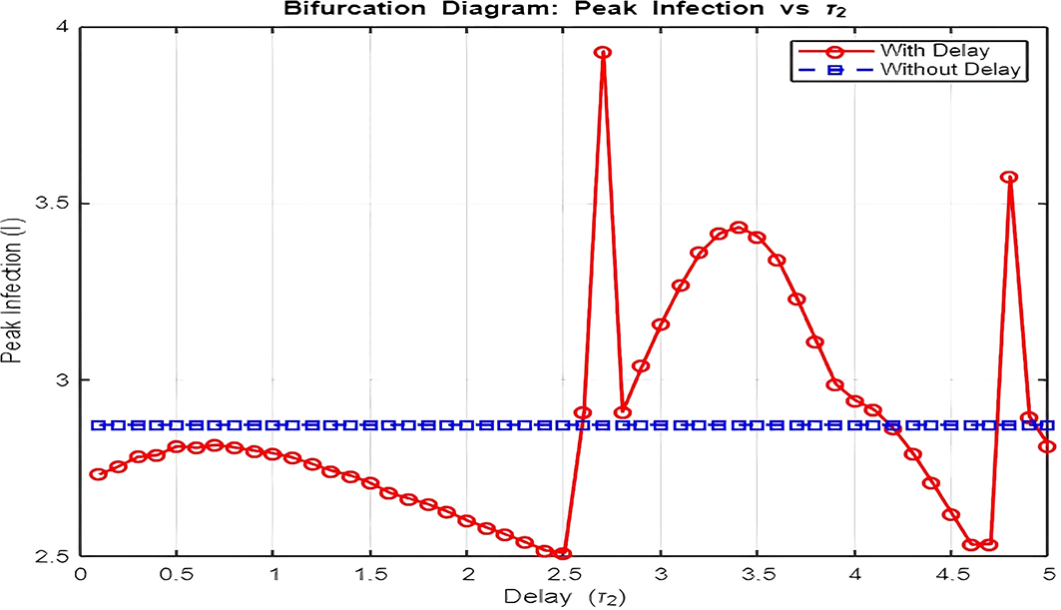
Lambda = 1.30; mu = 0.1; beta = 0.5; delta = 0.2; rho = 0.1; c = 0.7; tau = 0.6; tau2 = 2.5; m = 0.05; mu2 = 0.1; S0 = 5; I0 = 1; R10 = 0; R20 = 0; tspan = [0, 300].

**Figure 17.**
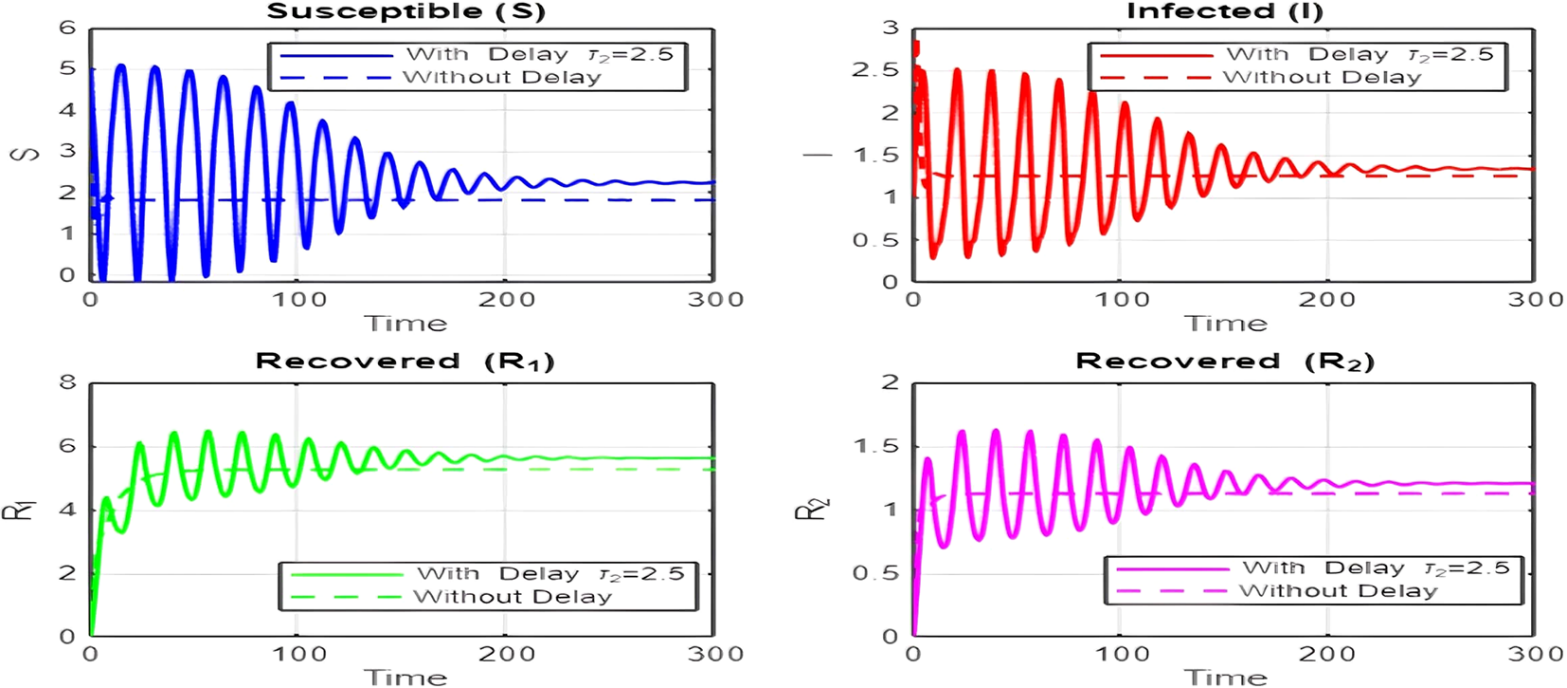
Lambda = 1.30; mu = 0.1; beta = 0.5; delta = 0.2; rho = 0.1; c = 0.7; tau = 0.6; tau2 = 2.5; m = 0.05; mu2 = 0.1; S0 = 5; I0 = 1; R10 = 0; R20 = 0; tspan = [0, 300].

## 8. Conclusion

This study provides a thorough analysis of the Ebola transmission dynamics using a deterministic SIRR model that incorporates key features such as nonlinear incidence rates and delayed recovery. By examining the stability of disease equilibria and the role of the basic reproduction number ( $R_{0}$ ), we have highlighted critical factors that influence the onset and persistence of outbreaks. The sensitivity analysis and bifurcation analysis, along with numerical simulations, underscore the importance of timely interventions and targeted strategies to reduce transmission, limit susceptible populations, and improve recovery rates. These insights offer valuable guidance for public health policy, particularly in managing outbreaks by identifying threshold conditions and critical parameters that determine the trajectory of disease spread. Overall, our findings contribute to a deeper understanding of Ebola dynamics and demonstrate the importance of considering delays in disease models to capture the complexities of real-world transmission.

## Ethics and consent

This study did not involve human participants or personal data; therefore, ethical approval and consent were not required.

## Data Availability

As outlined in our manuscript, the present study is purely theoretical in scope, focusing on conceptual development and does not involve the generation or analysis of empirical data.
