# Supplementary material for: Modeling and Analysis of SIRR Model (Ebola Transmission Dynamics Model) with Delay Differential Equation
Source: F1000Res. 2025 Sep 2;14:857. [Version 1] doi: 10.12688/f1000research.168361.1 (PMC12441668; doi:10.12688/f1000research.168361.1)
Supplement: Peer review history

# Reviewer report: Reviewer response for version 1

**DOI:** 10.5256/f1000research.185542.r412986

**Published:** 2025-09-22

**Authors:** Francis Oketch Ochieng

**Affiliations:** 1 HEALTH, International Livestock Research Institute, Nairobi, Kenya

The manuscript's proposed model is a solid mathematical framework for understanding the dynamics of Ebola virus disease (EVD) using delay differential equations (DDEs). It effectively captures several key aspects of Ebola transmission; and it utilizes key mathematical concepts such as stability, sensitivity, bifurcation, etc. Overall, the study is interesting and falls within the Journal's scope. However, some revisions would improve the quality of the paper. Its practical application could be enhanced by validating model’s predictions with real data on Ebola incidences and calibrating model parameters so that the model output trends is fitted on real data and considering heterogeneous mixing of individuals in the human population.

 What is the difference between this current study and (refer to 1) ??

**Specific Comments:**

1. **Abstract:** This section is poorly written. The abstract effectively summarizes the study’s focus but lacks quantitative results. Including a few key findings with numerical results will strengthen the abstract’s impact. Revise the abstract by keeping the following points in front.

1. **Background** of the presented problem.
2. **Aim** and **methodology** should be clearly defined, as it is not easy to understand it in the present form that the authors are going to discuss and which methodology adopted to acquire the model results.
3. The **key findings** of the study and some **quantitative results** must be added to the abstract. What value did you obtain for R _0_? Also include quantitative results on sensitivity analysis e.g., increase/decreasing progression rate (or environmental transmission coefficient or natural death rate) by x% leads to y% decrease/increase in R _0_.
4. **Applications** perspectives: one or two sentences which explain that in which field the acquired results would be beneficial; and explain how your results will inform policy decisions.
5. Check the correct tense to be used in an abstract.

 

1. **Keywords: **

- Avoid keywords that are already in the title of the paper.

 

1. **Introduction:** This section is poorly written. It is too short and lacks clarity. Some paragraphs are too long while others are too short.

- The literature review section should be improved, it should be dedicated to present critical analysis of state-of-the-art related work to justify the objective of the study. Also, critical comments should be made on the results of the cited works.

- The introduction should include a novelty paragraph describing previous research, potential research gaps, the authors' specific problem focus, and innovation. Highlight the study's main novelty in the last paragraph. Discuss how this model advances current understanding and applications in real world clinical scenarios. Provide a clearer statement of the research gap and how this model specifically addresses it.
- The Introduction should be improved by considering the related literature and should point out the motivation to the paper. It should make a compelling case for why the study is useful along with a clear statement of its novelty or originality by providing relevant information and providing answers to basic questions.

- I recommend revising the introduction Section to provide a concise but comprehensive overview of the background and recent progress in the field, leading to the motivation for the study.
- The authors should check the following articles to gain insight on how to write the introduction section of a manuscript:
  - (refer to 2,3,4)

 

1. **Methodology:**

- Your model should be abbreviated as SEIR not SIRR. Rephrase.
- Ensure the parameter values in Table 1 are within biologically realistic ranges. Also add the sources of these values.
- The sensitivity analysis of the basic reproduction number with respect to the model parameters should also state to what extent do the parameters influence the basic reproduction number. Use quantitative results in your explanations, not just stating the qualitative results.
- What exact numerical value of the basic reproduction number did you actually obtain from the computations? Please report that in your work. This threshold is very important to determine whether the disease will persist or die out in the human population.

1. **Numerical simulation:**

- What are the sources of the initial values used in the model simulations?
- Why did the authors randomly select the values of the parameters in this study? The choice of values of model parameters must be supported by biological facts. Consider estimating parameters that are not available in the literature, don’t just choose them randomly.

1. **Results:**

- This section lacks cited literature, which could help the reader directly understand the authors' contribution and originality of this work. More specifically, how do these findings align with existing literature, and what are the significant gaps in understanding that your work has addressed?
- The discussion on the results presented in graphs and tables should be improved significantly. A reasonable biological interpretation/explanation should be provided for the observed trends, not only report what is seen in the graphs and tables.
- A deeper discussion about the numerical results would be of benefit to potential readers of the manuscript‎. ‎

1. **Validation:**

- No attempt has been made to validate the results reported in this study with published sources. Therefore, the validity of these results are questionable. Can the authors explain this? It would be excellent if the importance of this issue was validated by detailed research and thoroughly documented data.
- Quantitative comparison with results from literature would enhance the impact of your findings. Also consider validating with real data on Ebola incidences for a particular region or nation.

1. **Conclusion: **

- Findings presented in the Conclusion section read very generic. All findings in the conclusion should be supported by **quantitative data**. Our focus is on the author's quantitative description based on quantitative data, not on qualitative descriptions.
- What are the **limitations** of this study?
- Where are the **recommendations to policymakers** in terms of commercialization of the research findings. Explain how the general reader might apply the outcomes of this research. Are there practical applications for predicting patient responses or optimizing treatment protocols?
- Can the authors also suggest some **areas for future research** based on their study? Future directions should be included in the conclusion part of the manuscript.

1. **English Presentation:** The manuscript requires further revision to meet the journal's English language standards. Consider seeking professional editing assistance.

**General Comment to Authors**

 I kindly request that you provide a thoroughly revised manuscript along with a point-by-point response delineating how you addressed my general and specific comments; I meant precisely that. As to the point-by-point response, a revised text in different colors (colors) is necessary. However, it is insufficient to inform me that you modified any given issue; it is essential to answer specifically HOW you addressed/changed each issue in the response letter. Please understand that I must request another revision/rejection of your manuscript if the revisions are inadequate.

Is the work clearly and accurately presented and does it cite the current literature?

Yes

If applicable, is the statistical analysis and its interpretation appropriate?

Partly

Are all the source data underlying the results available to ensure full reproducibility?

No

Is the study design appropriate and is the work technically sound?

Yes

Are the conclusions drawn adequately supported by the results?

No

Are sufficient details of methods and analysis provided to allow replication by others?

Yes

Reviewer Expertise:

Infectious Disease Modelling

I confirm that I have read this submission and believe that I have an appropriate level of expertise to state that I do not consider it to be of an acceptable scientific standard, for reasons outlined above.

## References

[1] https://pmc.ncbi.nlm.nih.gov/articles/PMC12441668/.

[2] : Mathematical Modeling of Tuberculosis Transmission Dynamics With Reinfection and Optimal Control. *Engineering Reports* .2025;7(1) : 10.1002/eng2.13068 10.1002/eng2.13068

[3] : Modeling tuberculosis dynamics with vaccination and treatment strategies. *Scientific African* .2025;28: 10.1016/j.sciaf.2025.e02647 10.1016/j.sciaf.2025.e02647

[4] : SEIRS model for TB transmission dynamics incorporating the environment and optimal control. *BMC Infectious Diseases* .2025;25(1) : 10.1186/s12879-025-10710-2 10.1186/s12879-025-10710-2 40205340 PMC11983798

# Reviewer report: Reviewer response for version 1

**DOI:** 10.5256/f1000research.185542.r412989

**Published:** 2025-09-16

**Authors:** Hakeem Adekunle, Kayode Okunola

**Affiliations:** 1 Mathematics and Statistics, Georgia State University, Atlanta, Georgia, USA

The author states that they have developed and analyzed a novel deterministic SEIRR model that captures the complex transmission dynamics of Ebola by combining nonlinear incidence rates with a delay differential equation. While the methodology appears scientifically sound, Section 2 (“Model Formulation”) notes that the model was formulated by examining the SEIRR model presented in Ref. 39. This creates confusion and should be clarified to ensure consistency between the stated SEIRR model and the referenced SEIRR model.

 In Section 2.3 (“Boundedness of the Model”), the theorem presented is important to the study. However, it would be more appropriate to simply state or declare the theorem in the main text and move the full proof to an appendix for better readability.

 The authors performed sensitivity analysis, bifurcation analysis, and numerical simulations, through which they identified critical factors influencing the onset and persistence of outbreaks. A key finding of the study is the importance of incorporating delays into disease models to better capture the complexities of real-world transmission dynamics.

Is the work clearly and accurately presented and does it cite the current literature?

Yes

If applicable, is the statistical analysis and its interpretation appropriate?

Yes

Are all the source data underlying the results available to ensure full reproducibility?

No source data required

Is the study design appropriate and is the work technically sound?

Yes

Are the conclusions drawn adequately supported by the results?

Yes

Are sufficient details of methods and analysis provided to allow replication by others?

Partly

Reviewer Expertise:

Biostatistics, Machine learning, epidemiology, Causal Inference, Measurement error, Bayesian, meta-analysis.

We confirm that we have read this submission and believe that we have an appropriate level of expertise to confirm that it is of an acceptable scientific standard.

# Reviewer report: Reviewer response for version 1

**DOI:** 10.5256/f1000research.185542.r412990

**Published:** 2025-09-16

**Authors:** Peter Olatunji

**Affiliations:** 1 Adekunle Ajasin University, Akungba, Ondo, Nigeria

The study develops and analyzes a SIRR model for Ebola transmission dynamics as well as incorporating nonlinear incidence rates and delay differential equations to capture the latent period before infectiousness. The authors demonstrate how delays significantly affect outbreak persistence and oscillatory epidemics by deriving the basic reproduction number​, performing stability and bifurcation analyses, and running numerical simulations.

 The work is clearly structured with a logical flow from model formulation to analysis and interpretation.

 The study is primarily theoretical and does not include empirical statistical testing, but its bifurcation and sensitivity analyses are mathematically appropriate.

 The study explicitly states that it is purely theoretical and does not rely on empirical data. Thus, full reproducibility is ensured through the provided mathematical formulations and analysis.

 The conclusions directly follow from the analytical and simulation results, showing how delays and isolation affect outbreak persistence

Is the work clearly and accurately presented and does it cite the current literature?

Yes

If applicable, is the statistical analysis and its interpretation appropriate?

Not applicable

Are all the source data underlying the results available to ensure full reproducibility?

No source data required

Is the study design appropriate and is the work technically sound?

Yes

Are the conclusions drawn adequately supported by the results?

Yes

Are sufficient details of methods and analysis provided to allow replication by others?

Yes

Reviewer Expertise:

Scientific Computing, Biomathematics, Statistical Computing, Numerical Analysis

I confirm that I have read this submission and believe that I have an appropriate level of expertise to confirm that it is of an acceptable scientific standard.
